# Astragaloside IV Alleviates Fructose-Induced Intestinal Metabolic Senescence by Targeting Ketohexokinase Asn261/Ala226 to Preserve Intestinal Stem Cell Homeostasis

**DOI:** 10.1021/acscentsci.5c00726

**Published:** 2025-07-29

**Authors:** Qifang Wu, Yingna Li, Yunyun Zhao, Ruifen Zhang, Jingyang Tong, Chunlei Ji, Yiming Zhao, Mingjiang Wu, Xiaosheng Jin, Dandan Wang, Haibin Tong, Liwei Sun, Fangbing Liu

**Affiliations:** † Research Center of Traditional Chinese Medicine, College of Traditional Chinese Medicine, 159345 Changchun University of Chinese Medicine, Changchun 130021, China; ‡ Northeast Asian Institute of Traditional Chinese Medicine, Changchun University of Chinese Medicine, Changchun 130021, China; § College of Life and Environmental Science, 26495 Wenzhou University, Wenzhou 325035, China; ∥ Department of Endocrinology and Metabolism, 159346 Affiliated Hospital to Changchun University of Chinese Medicine, Changchun 130021, China; ⊥ Department of Gastroenterology, The Third Affiliated Hospital of Wenzhou Medical University, Wenzhou 325200, China

## Abstract

Excessive fructose intake drives intestinal aging and impairs intestinal stem cell (ISC) function, yet effective therapeutic interventions remain elusive. Astragaloside IV (AS-IV), a natural saponin from *Astragalus membranaceus*, has been widely recognized for its antiaging, anti-inflammatory, and gut-protective properties. Here, we revealed that AS-IV alleviates fructose-induced intestinal metabolic senescence via direct inhibition of ketohexokinase (KHK), the key rate-limiting enzyme in fructose metabolism. Molecular docking and site-directed mutagenesis identified Asn261 and Ala226 as distinct binding sites for AS-IV on KHK, with Asn261 also serving as a critical catalytic residue that is essential for KHK activity. Mutation at Asn261 abolished KHK enzymatic function, reduced the accumulation of fructose-derived metabolites such as palmitic acid and ceramide, and thereby prevented fructose-induced ISC cycle arrest. AS-IV’s therapeutic efficacy was validated across *Drosophila*, murine intestinal organoids, and mice, where treatment consistently reversed high-fructose-induced intestinal metabolic senescence phenotypes, restored ISC proliferation, and preserved ISC homeostasis. These findings indicate that KHK is a previously unrecognized molecular target of AS-IV and reveal a conserved mechanism by which AS-IV modulates fructose metabolism to interfere with gut aging. Our results highlight its therapeutic potential in treating fructose-driven intestinal aging and associated metabolic disorders.

## Graphical abstract

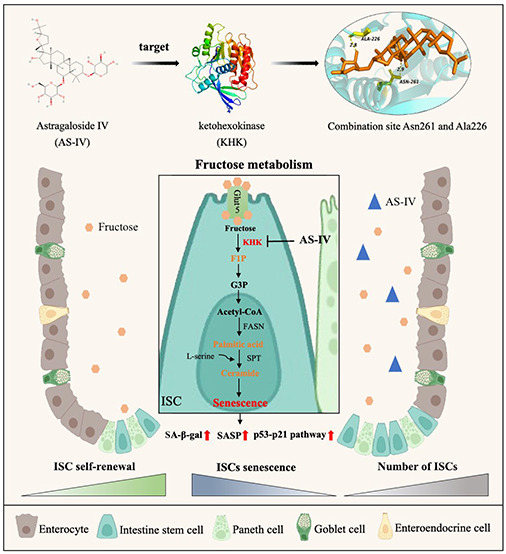

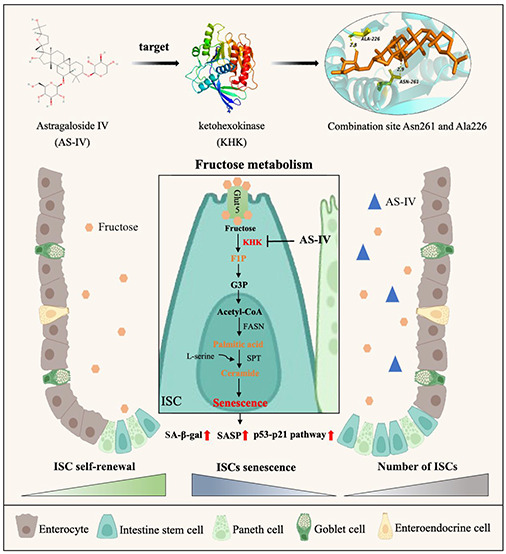

## 1 Introduction

Metabolic senescence refers to the aging phenomena associated with an impaired metabolic function and abnormal metabolic processes. It is controlled by various interrelated factors, including genetic predisposition, environmental influences, and lifestyle,^1^ with diet emerging as one of the most significant contributing factors. Recent studies have linked excessive fructose consumption with increased production of senescence-associated secretory phenotype (SASP) and cellular senescence,^2–4^ both of which are hallmarks of aging. Fructose has emerged as a key contributor to metabolic senescence due to its unique metabolic pathway. Initially, fructose was believed to be metabolized exclusively in the liver after absorption through the portal vein,^5^ recent evidence highlights the small intestine as a critical site for fructose metabolism.^6^ Fructose-derived metabolites, including palmitic acid and ceramide, impair intestinal epithelial cell function, compromising barrier integrity and nutrient absorption.^7–9^ These disruptions contribute to intestinal metabolic senescence over time. Furthermore, research has reported that short-term excessive fructose intake can directly alter the metabolism of intestinal crypt cells, inhibiting the regenerative proliferation of ISC and transit-amplifying (TA) cells while also impairing their ability to repair after damage.^10^ Therefore, excessive fructose consumption accelerates metabolic senescence by disrupting intestinal function and impairing ISC regeneration, necessitating the exploration of strategies to mitigate these effects.

ISCs play a central role in maintaining epithelial integrity and barrier function by continuously self-renewing and differentiating to replenish intestinal epithelial cells, which undergo turnover every four to 6 days.^11^ This high turnover demands that ISCs maintain a balance between proliferation and differentiation to preserve the integrity of the villi and crypts, which are essential for nutrient absorption. Previous studies have indicated that aging results in reduced size and density of villi and crypts in mice, likely associated with inhibited ISC proliferation during the aging process.^12^ Therefore, the aging of ISCs is characterized by stem cell exhaustion, with damage to ISC function leading to a decline in their self-renewal capacity and ultimately resulting in intestinal epithelial dysfunction. Given the critical role of ISCs in intestinal aging and their vulnerability to metabolic disruptions, it is crucial to identify effective therapeutic strategies that can mitigate fructose-induced metabolic senescence and promote ISC function.

Astragaloside IV (AS-IV), a natural saponin and major bioactive component of *Astragalus membranaceus*, has been reported to have a variety of pharmacological activities, including immunomodulatory, antioxidative, anti-inflammatory, antidiabetic, and antiaging properties.^13,14^ Numerous studies have demonstrated that AS-IV can significantly reduce inflammation and increase intestinal epithelial permeability.^15^ Moreover, AS-IV mitigates radiation-induced brain cellular senescence by regulating the p53-p21 and p16-RB signaling pathways in aging.^16^ AS-IV has also been reported to mitigate metabolic disturbances induced by fructose consumption, suggesting its potential as a therapeutic agent for metabolic dysfunction.^17^ However, the ability of AS-IV to alleviate intestinal metabolic senescence remains unclear and requires further investigation. Therefore, this study aimed to investigate the protective effects of AS-IV against fructose-induced intestinal metabolic senescence, its molecular targets, and the underlying regulatory mechanisms. These findings not only contribute to the understanding of AS-IV’s antiaging mechanisms but also provide insights into its potential clinical applications for preventing age-related diseases associated with excessive fructose intake.

## 2 Results

### 2.1 AS-IV Alleviates Fructose-Induced Metabolic Senescence in IEC-6 Cells

To investigate the effect of AS-IV on fructose-induced metabolic senescence in IEC-6 cells, we first characterized senescence under high-fructose conditions. IEC-6 cells were treated with 5 mM, 10 mM, or 20 mM fructose for 24 or 48 h. SA-β-gal staining showed that 10 mM fructose treated for 48 h increased SA-β-gal activity (Figure S1A and S1C), which was consistent with an increased mRNA level of *Il6*, as well as up-regulated expression of SASP markers (*Il1b* and *Tnf*) (Figure S1B and S1D). These changes observed in these IEC-6 cells were not caused by osmotic pressure, since IEC-6 cells cultured for 48 h in 10 mM mannitol or glucose showed no sign of increased SA-β-gal activity and *Il6*, *Il1b*, and *Tnf* mRNA levels (Figure S1E and S1F). These findings suggest that high-fructose conditions contribute to metabolic senescence in IEC-6 cells.

Following the establishment of fructose-induced senescence in IEC-6 cells, the effect of AS-IV on senescence was investigated by cotreating the cells with 10 mM fructose (HFru) and 1, 5, or 10 μM AS-IV. Treatment with 5 μM AS-IV suppressed fructose-induced SA-β-gal activity (Figure S2A and Figure 1A). IEC-6 cells treated with fructose and AS-IV also displayed lower *Il6*, *Il1b*, and *Tnf* mRNA levels than those treated with fructose alone (Figure S2B and Figure 1B-D). Preliminary data suggest that high fructose activates the p53-p21 signaling pathway, leading to intestinal cell cycle arrest (unpublished data). Consistently, flow cytometry analysis revealed that fructose-treated IEC-6 cells exhibited increased cell cycle arrest, primarily at the G0/G1 phase, whereas cotreatment with fructose and AS-IV largely mitigated this effect (Figure 1E and 1F). The levels of p53 and p21 are commonly used to identify senescent cells.^18^ Fructose-treated IEC-6 cells showed increased *Tp53*, *Cdkn1a*, and *Rb1* mRNA levels and decreased *Cdk4* and *E2f1* mRNA levels, whereas these effects were absent in cells treated with HFru + AS-IV (Figure 1G). Protein analysis further confirmed that AS-IV inhibited p53 and p21 expressions while activating CDK4 (Figure 1H and 1I). These findings indicate that AS-IV effectively alleviates fructose-induced metabolic senescence in IEC-6 cells.

**Figure 1:**
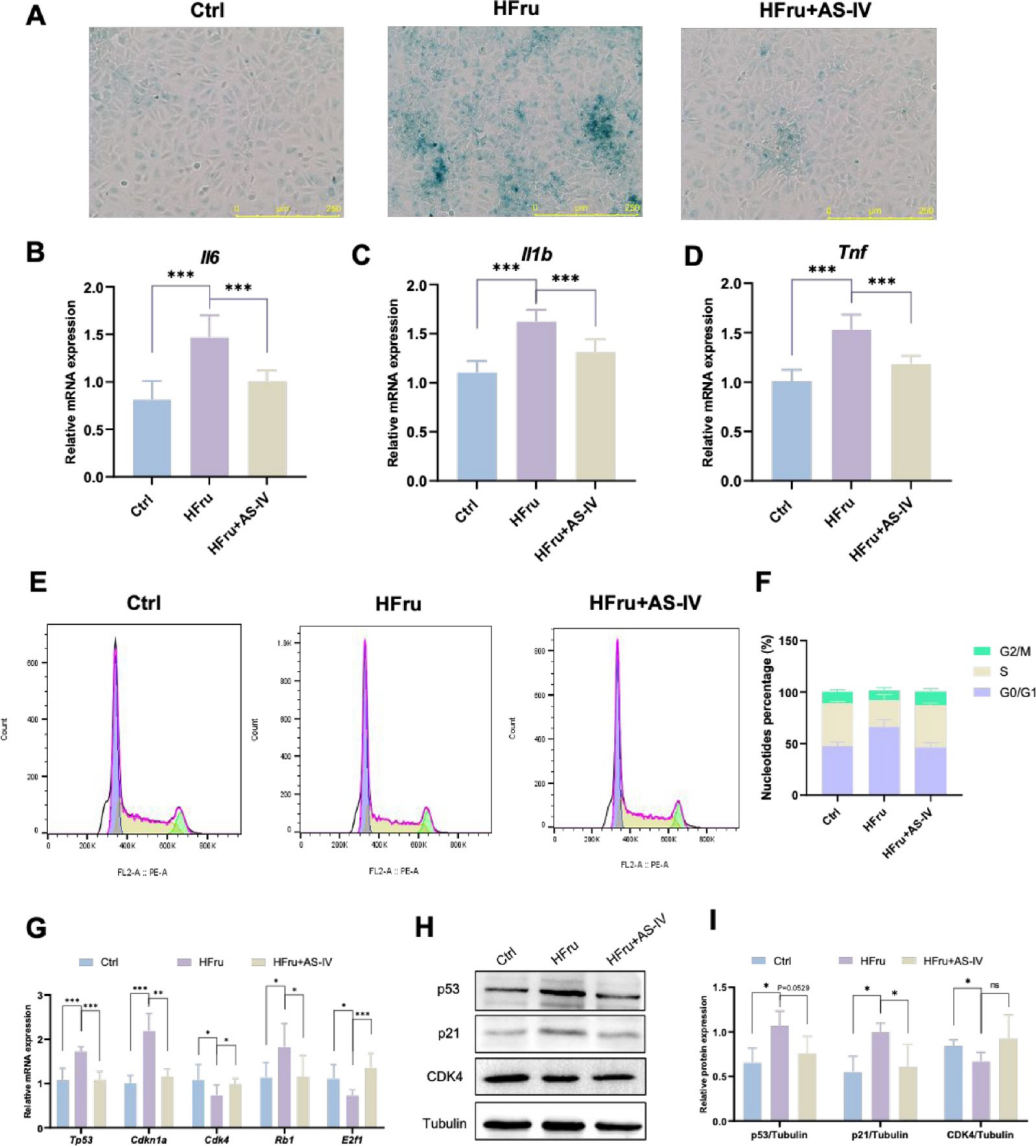
AS-IV alleviates fructose-induced metabolic senescence in IEC-6 cells. (A) SA-β-gal staining of IEC-6 cells. (B-D) RT-qPCR analysis of inflammatory gene expression associated with the SASP, including *Il6, Il1b*, and *Tnf*, in IEC-6 cells. (E-F) Cell cycle analysis by flow cytometry, with quantitative analysis of the percentage of cells in different phases. (G) RT-qPCR analysis of cell cycle-related gene expression (*Tp53, Cdkn1a, Cdk4, Rb1*, and *E2f1*) in IEC-6 cells. (H-I) Representative Western blots and quantitative analysis of the indicated protein expression in IEC6 cells. Data are expressed as the mean ± SD (*n* = 3). ‘*’, ‘**’, ‘***’, ‘ns’ indicate significant differences at *P* < 0.05, *P* < 0.01, *P* < 0.001, and no significance levels, respectively.

### 2.2 AS-IV Alleviates Fructose-Induced Metabolic Senescence in *Drosophila* Gut

Given the protective role of AS-IV observed in IEC-6 cells under fructose-induced stress, we next investigated whether similar effects could be observed in an *in vivo Drosophila* model exposed to a high-fructose diet (HFruD). These flies exhibited increased SA-β-gal activity in the gut compared to control (Ctrl) flies. On the other hand, flies fed a mannose diet, used as an isosmotic control, exhibited SA-β-gal activity levels similar to those of the Ctrl group. However, supplementation with 25 μM, 50 μM, or 100 μM AS-IV suppressed fructose-induced SA-β-gal activity in the gut, with 100 μM AS-IV being the most effective (Figure S3A and Figure 2A). Flies treated with HFruD + AS-IV also displayed reduced *Upd3* (a homologue of *Il6*) and *Eiger* (a homologue of *Tnf*) mRNA levels in the gut (Figure S3B and Figure 2B) and decreased *P53*, *Dap*, and *Rbf* mRNA levels, along with increased *Cdk4* and *E2f1* mRNA levels, leading to alleviated cell cycle arrest (Figure 2C). ISCs are crucial for tissue homeostasis in the intestine and are especially vulnerable to exhaustion, a key feature of aging.^19^ To further evaluate the effects of AS-IV on ISC senescence, the *esg* ^*ts*^ system (*esg-Gal4; UAS-GFP; tub-Gal80* ^*ts*^) was used to assess the number and self-renewal capacity of ISCs in the *Drosophila* gut (Figure 2D). Flies treated with AS-IV exhibited a smaller reduction in ISC numbers caused by the high-fructose diet (Figure 2E) and showed an increase in the number of PH3^+^ cells (Figure 2F), indicative of actively dividing cells. These findings highlight the protective role of AS-IV against cellular senescence induced by a high-fructose diet, particularly in ISC dynamics and proliferation.

**Figure 2:**
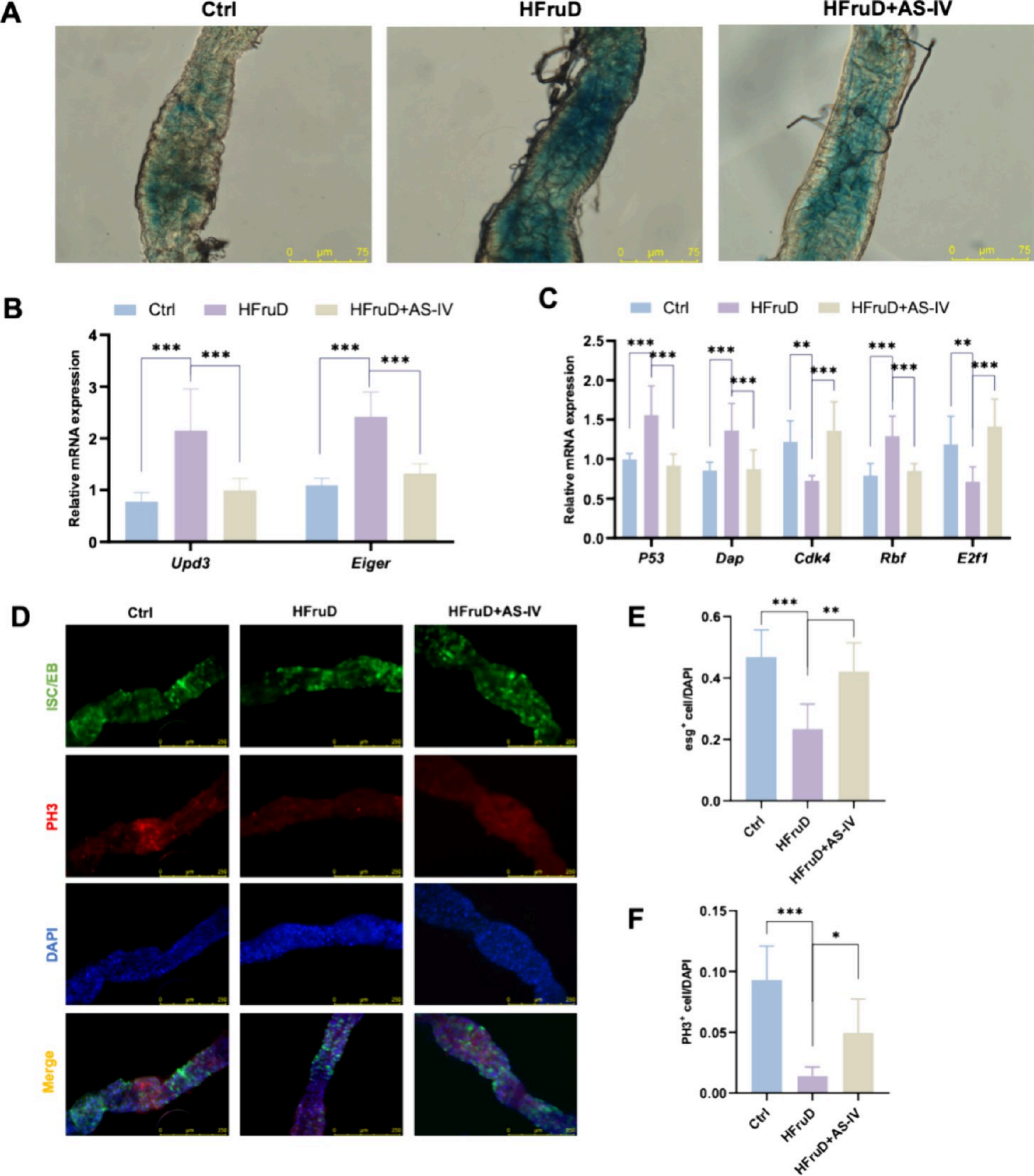
AS-IV alleviates fructose-induced metabolic senescence in the *Drosophila* intestine. (A) SA-β-gal staining of the *Drosophila* gut. (B) RT-qPCR analysis of inflammatory gene expression associated with the SASP, including *Upd3* and *Eiger*, in the *Drosophila* gut. (C) RT-qPCR analysis of cell cycle-related gene expression (*P53, Dap, Cdk4, Rbf*, and *E2f1*) in the *Drosophila* gut. (D) Representative immunofluorescence images of the *Drosophila* gut (*esg-Gal4; UAS-GFP; tub-Gal80* ^*ts*^). Samples were costained with DAPI (blue) and anti-PH3 (red). Scale bar, 250 μm. (E) Quantification of *esg* ^*+*^ cells. (F) Quantification of *PH3* ^*+*^ cells. Data are expressed as mean ± SD (*n* = 3). ‘*’, ‘**’, ‘***’, ‘ns’ indicate significant differences at *P* < 0.05, *P* < 0.01, *P* < 0.001, and no significance levels, respectively.

### 2.3 AS-IV Alleviates Fructose-Induced ISC Senescence Determined by Intestinal Organoid

To further elucidate the role of AS-IV in regulating ISC renewal, we assessed the ability of isolated epithelial crypts to form clonogenic organoids as an *ex vivo* assay of ISC function. Fructose exposure significantly impaired organoid self-renewal capacity and inhibited growth (Figure 3A). AS-IV promoted organoid growth and improved the self-renewal capacity. Fructose-treated organoids exhibited increased *Il6*, *Il1b*, *Tnf*, *Trp53*, *Cdkn1a*, and *Rb1* mRNA levels (Figure 3B and 3C) and reduced *Cdk4* and *E2f1* mRNA levels (Figure 3C), along with increased p53 and p21 and decreased CDK4 protein levels (Figure 3E and 3F) compared with the control. These alterations were largely reversed by AS-IV intervention. To evaluate the effect of AS-IV on ISC proliferation, immunostaining for Ki67 demonstrated a significant reduction in the proliferative activity in organoids exposed to high fructose. However, AS-IV treatment mitigated this effect, restoring proliferation (Figure 3D). Furthermore, AS-IV effectively increased the density of OLFM4-positive stem cells in high-fructose-treated intestinal organoids (Figure 3G). AS-IV also up-regulated the expression of ISC markers, including *Olfm4*, *Lgr5*, and *Ascl2*, thus mitigating the fructose-induced reduction in ISC numbers (Figure 3H). These findings indicate that AS-IV promotes intestinal organoid growth and ISC proliferation, while alleviating fructose-induced ISC senescence.

**Figure 3:**
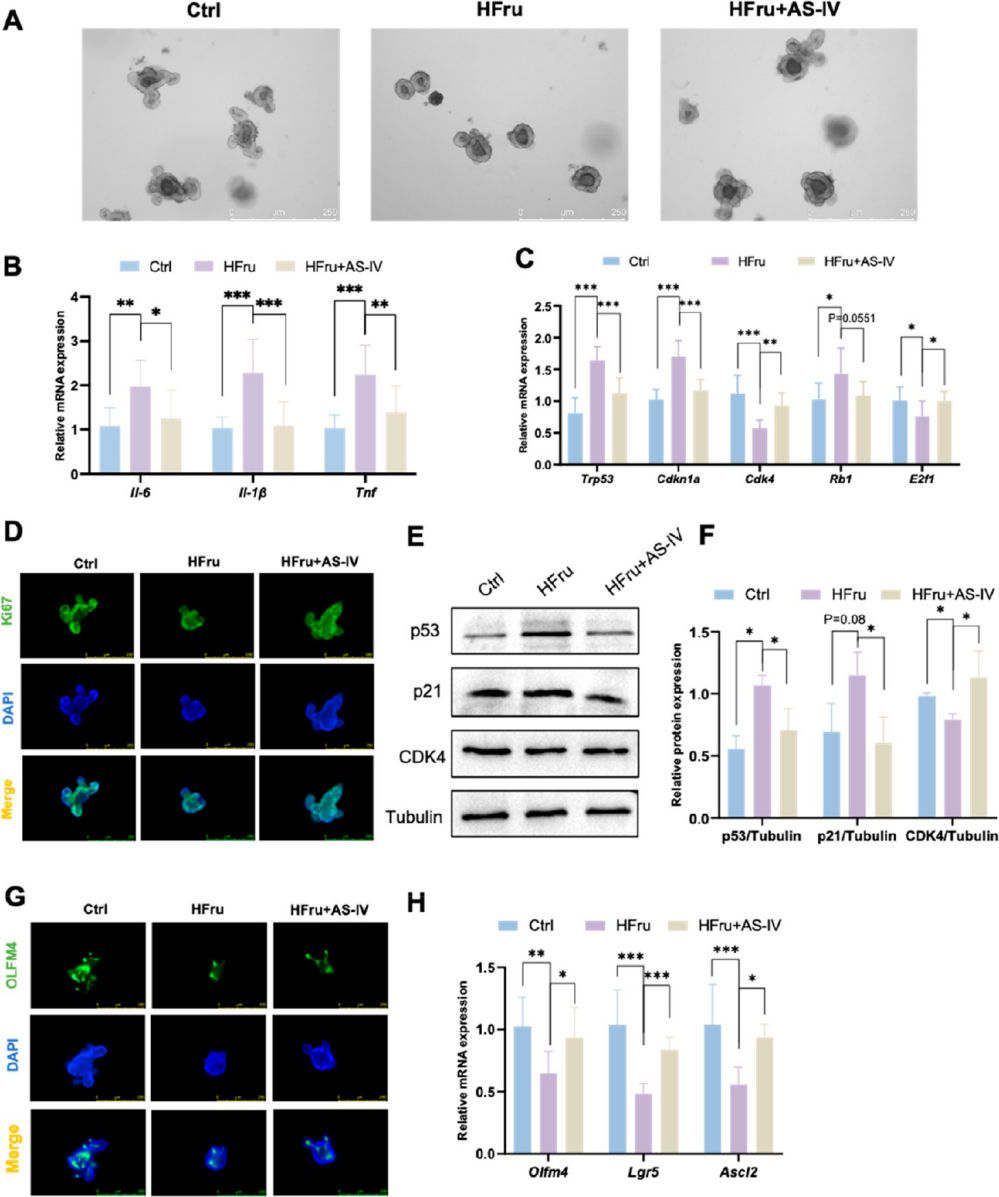
AS-IV stimulates intestinal organoids growth. (A) Representative images of intestinal organoids. (B) RT-qPCR analysis of inflammatory gene expression associated with the SASP, including *Il6, Il1b*, and *Tnf*, in intestinal organoids. (C) RT-qPCR analysis of cell cycle-related gene expression (*Trp53, Cdkn1a, Cdk4, Rb1*, and *E2f1*) in intestinal organoids. (D) Representative immunofluorescence images of Ki67 (green) in intestinal organoids. (E-F) Representative Western blots and quantitative analysis of indicated protein expression in intestinal organoids. (G) Representative immunofluorescence images of OLFM4 (green) in intestinal organoids. (H) RT-qPCR analysis of intestinal stem cell marker expression (*Olfm4, Lgr5*, and *Ascl2*) in intestinal organoids. Data are expressed as mean ± SD (*n* = 3). ‘*’, ‘**’, ‘***’ indicate significant differences at *P* < 0.05, *P* < 0.01, and *P* < 0.001 levels, respectively.

### 2.4 AS-IV Inhibits the Accumulation of Intestinal Fructose Metabolites by Suppressing KHK Activity

To further explore the mechanism by which AS-IV attenuates fructose-induced ISC senescence, we next investigated whether its protective effects are mediated through the modulation of fructose. Fructose is metabolized to fructose-1-phosphate (F1P) and glyceraldehyde-3-phosphate, which enter the glycolytic pathway to produce acetyl-CoA. Acetyl-CoA is then used for the synthesis of fatty acids such as palmitic acid and ceramide (Figure 4A). The accumulation of ceramide is known to induce cellular senescence. To determine whether AS-IV alleviates cellular senescence by inhibiting fructose metabolism, palmitic acid and ceramide levels were measured in high-fructose-treated intestinal organoids. AS-IV treatment reduced the accumulation of both palmitic acid (Figure 4B) and ceramide (Figure 4C) in high-fructose-treated organoids, suggesting that it may prevent the buildup of senescence-associated metabolites. To further investigate how AS-IV regulates fructose metabolism, intestinal organoids were treated separately with palmitic acid and ceramide. However, AS-IV did not rescue the growth inhibition or restore ISC numbers and proliferation induced by palmitic acid or ceramide (Figure 4D-I), suggesting that AS-IV primarily exerts its effects at an upstream stage of the fructose metabolic pathway.

**Figure 4:**
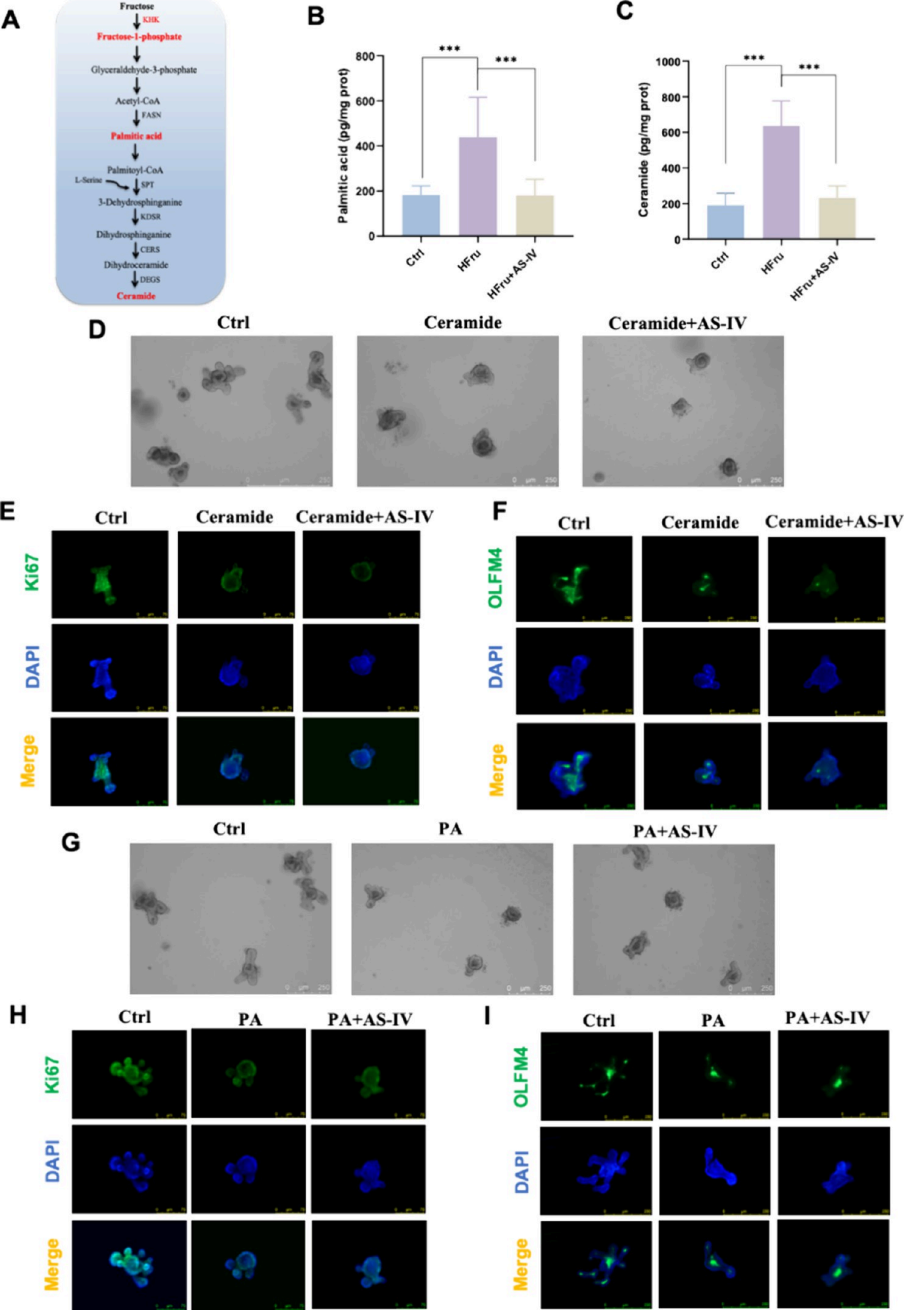
AS-IV reduces fructose metabolite accumulation of palmitic acid and ceramide in intestinal organoids. (A) Fructose metabolism, fatty acid biosynthesis, and ceramide *de novo* synthesis pathways. (B) Palmitic acid content in intestinal organoids. (C) Ceramide content in intestinal organoids. (D) Representative images of intestinal organoids treated with ceramide or ceramide with AS-IV (ceramide+AS-IV). (E) Representative immunofluorescence images of Ki67 (green) in intestinal organoids under ceramide and ceramide+AS-IV treatments. (F) Representative immunofluorescence images of OLFM4 (green) in intestinal organoids under ceramide and ceramide+AS-IV treatments. (G) Representative images of intestinal organoids treated with palmitic acid (PA) or palmitic acid with AS-IV (PA+AS-IV). (H) Representative immunofluorescence images of Ki67 (green) in intestinal organoids under PA and PA+AS-IV treatments. (I) Representative immunofluorescence images of OLFM4 (green) in intestinal organoids under PA and PA+AS-IV treatments. Data are expressed as mean ± SD (*n* = 3). ‘*’, ‘**’, ‘***’ indicate significant differences at *P* < 0.05, *P* < 0.01, and *P* < 0.001 levels, respectively.

Subsequently, we further investigated whether AS-IV exerts its effects at an upstream stage of fructose metabolism, thereby preventing the accumulation of downstream metabolic byproducts. To examine this possibility, intestinal organoids were treated directly with F1P, the direct product of KHK. AS-IV treatment failed to rescue F1P-induced growth inhibition (Figure 5A) or ISC proliferation suppression (Figure 5B and 5C), indicating that AS-IV does not directly counteract the effects of F1P. KHK, a key rate-limiting enzyme in fructose metabolism. Given the essential role of KHK in this pathway, it was hypothesized that AS-IV alleviates fructose-induced metabolic senescence by inhibiting KHK activity. Indeed, both KHK activity measurements in organoids (Figure 5D) and an *in vitro* enzyme activity assay (Figure 5E) confirmed that AS-IV inhibits KHK activity, therefore mitigating the metabolic alterations that drive cellular senescence.

**Figure 5:**
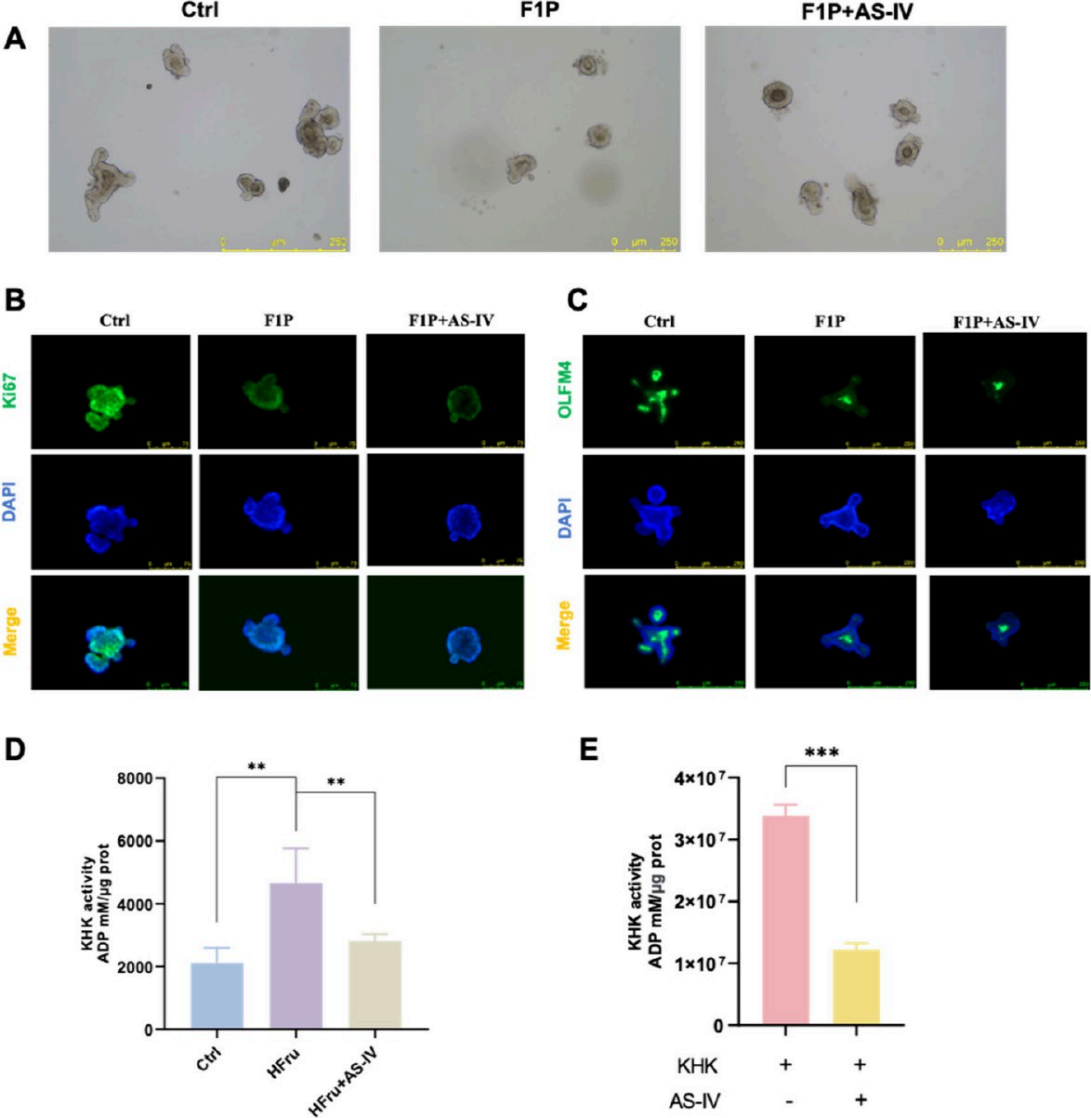
AS-IV inhibits KHK activity to reduce the accumulation of intestinal fructose metabolites. (A) Representative images of intestinal organoids treated with D-fructose 1-phosphate disodium salt (F1P) or F1P with AS-IV (F1P+AS-IV). (B) Representative immunofluorescence images of Ki67 (green) in intestinal organoids under F1P and F1P+AS-IV treatments. (C) Representative immunofluorescence images of OLFM4 (green) in intestinal organoids under F1P and F1P+AS-IV treatments. (D) KHK activity in intestinal organoids. (E) KHK activity inhibition assay. Data are expressed as mean ± SD (*n* = 3). ‘*’, ‘**’, ‘***’ indicate significant differences at *P* < 0.05, *P* < 0.01, and *P* < 0.001 levels, respectively.

### 2.5 KHK Is the Potential Molecular Target of AS-IV

Based on AS-IV’s ability to inhibit KHK enzymatic activity, we proposed that KHK may act as a key molecular mediator through which AS-IV attenuates cellular senescence. To explore the potential direct interaction between AS-IV and KHK, CETSA and ITDRF_CETSA_ analyses were performed. CETSA (Figure 6A and 6B) and ITDRF_CETSA_ (Figure 6C and 6D) demonstrated that AS-IV treatment effectively protected KHK from thermal degradation with thermal stabilization showing a dose-dependent relationship. Moreover, DARTS analysis revealed reduced proteolysis of KHK upon AS-IV treatment (Figure 6E and 6F). To provide direct evidence of the binding interaction between KHK and AS-IV, microscale thermophoresis (MST) was used to determine the binding affinity. MST, a biophysical technique that measures the thermophoretic displacement of fluorescent molecules in a temperature gradient, is widely used to determine dissociation constants (*K*d) for small compound-protein interactions. The results yielded a *K*d of 5.9251 μM (Figure 6G), indicating strong direct binding between AS-IV and KHK. These findings provide robust evidence that KHK is a molecular target of AS-IV.

**Figure 6:**
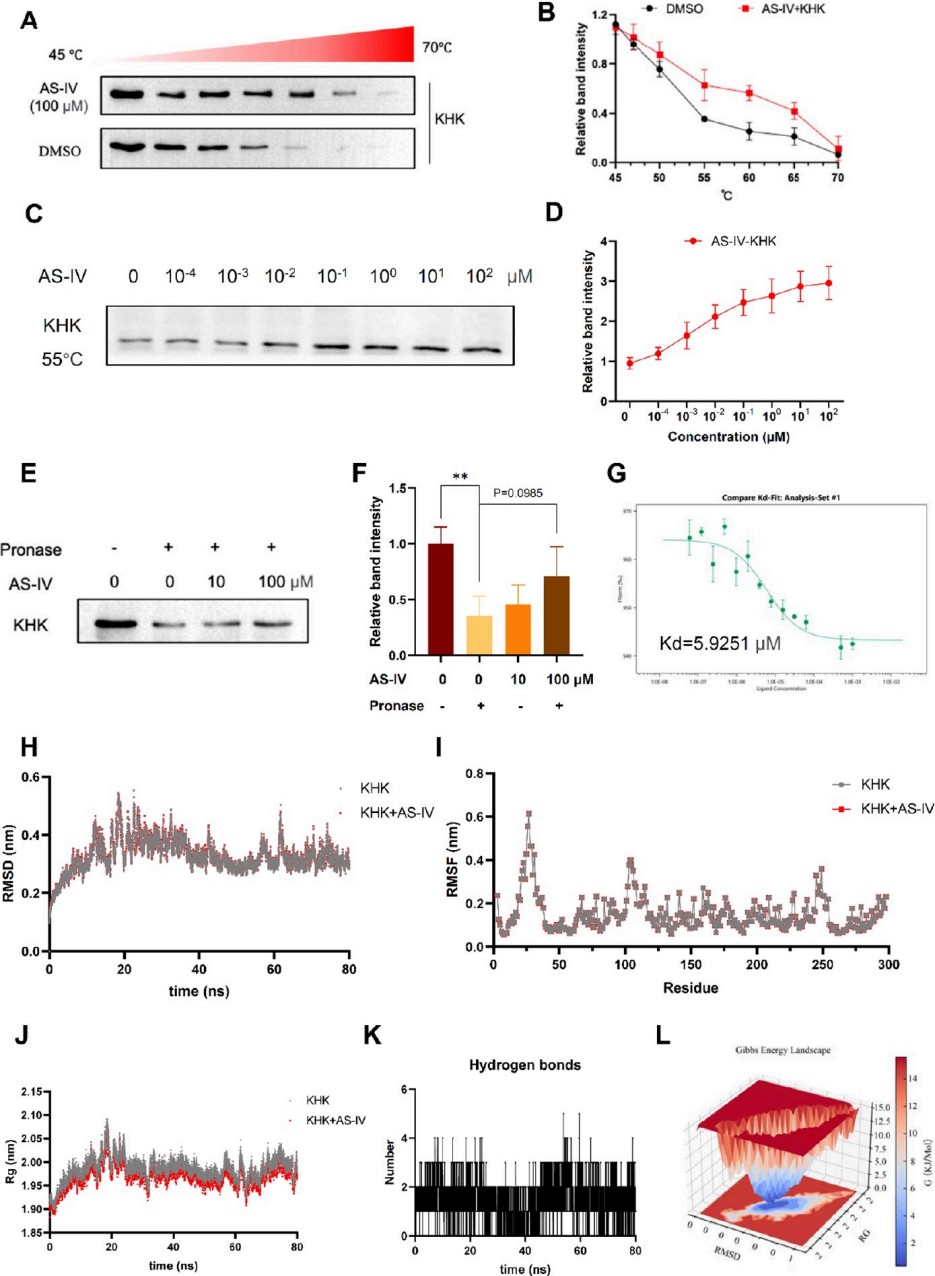
KHK is the potential target of AS-IV. (A, B) Cellular thermal shift assay (CETSA) assessing the binding between AS-IV and KHK at thermodynamic levels. (C, D) Isothermal dose–response fingerprint CETSA (ITDRF_CETSA_) assessing the binding between AS-IV and KHK at 55 °C. (E, F) Drug affinity responsive target stability (DARTS) assay showing that AS-IV increases KHK resistance to proteolysis. (G) Microscale thermophoresis (MST) analysis of AS-IV binding to KHK, with dissociation constants calculated from three independent replicates. (H) Root mean square deviation (RMSD) analysis over time based on molecular dynamics simulations. (I) Root mean square fluctuation (RMSF) calculated from molecular dynamics simulation trajectories. (J) Radius of gyration (Rg) plot derived from molecular dynamics simulations. (K) Hydrogen bond analysis based on molecular dynamics simulations. (L) Gibbs free energy landscape analysis. Data are expressed as mean ± SD (*n* = 3). ‘*’, ‘**’, ‘***’, ‘ns’ indicate significant differences at *P* < 0.05, *P* < 0.01, *P* < 0.001, and no significance levels, respectively.

Molecular dynamics simulations were then performed to investigate the dynamic behavior and stability of the AS-IV-KHK complex. The root-mean-square deviation (RMSD) values for the complex remained stable between 0.3 and 0.5 nm over a 20-80 ns simulation period, indicating no significant conformational changes (Figure 6H). The root-mean-square fluctuation (RMSF) of KHK in the presence of AS-IV was relatively low, with most amino acid residues displaying RMSF values below 0.3 nm, suggesting a stable protein structure (Figure 6I). The radius of gyration, a measure of the compactness of the protein’s three-dimensional structure, remained stable throughout the simulation, fluctuating between 1.95 and 2.05 nm, indicating a consistent conformation without significant expansion or contraction (Figure 6J). The number of hydrogen bonds fluctuated between 0 and 5 throughout the simulation, suggesting a dynamic hydrogen bond formation and dissociation process between AS-IV and KHK (Figure 6K). The Gibbs free energy landscape of the AS-IV–KHK complex revealed that the dark blue regions corresponded to the lowest energy state, representing the most stable conformation (Figure 6L). Overall, these findings confirm that AS-IV directly binds to KHK, stabilizing the protein and suppressing its enzymatic activity.

### 2.6 AS-IV Interacts with KHK via Asn261 and Ala226

Molecular docking was performed to identify the potential binding sites of AS-IV on KHK. The results indicated that AS-IV exhibited a strong binding affinity to KHK, with a binding energy of −10.8 kcal/mol, primarily driven by hydrogen bonds formed at Asn261 and Ala226 (Figure 7A). To further investigate the functional significance of these residues, site-directed mutagenesis was performed using the AlphaFold3 platform, replacing Asn261 and Ala226 with Ala261 and Gly226, respectively (N261A and A226G). Molecular docking analyses revealed a reduced binding affinity of AS-IV for the mutant KHK proteins with binding energies of −7.6 kcal/mol for both KHK^N261A^ and KHK^A226G^ (Figure S5A and S5B). When both mutations were introduced simultaneously (KHK^N261A, A226G^), the binding energy further decreased to −5.4 kcal/mol (Figure 7B). Furthermore, MST experiments showed that the *K*d of AS-IV for KHK^N261A^ and KHK^A226G^ were 75.613 and 55.383 μM (Figure S5C and S5D), respectively. Importantly, the double mutant protein exhibited a significantly reduced affinity with a *K*d of 264.46 μM (Figure 7C). Moreover, CETSA analysis revealed a decline in the thermal stability of the mutated proteins upon binding AS-IV (Figure 7D), confirming that Asn261 and Ala226 are key residues mediating AS-IV binding. Interestingly, *in vitro* enzymatic activity assays of KHK mutants revealed that KHK^A226G^ exhibited significantly reduced enzymatic activity, retaining only 1% of KHK activity, indicating partial functionality. Moreover, AS-IV could still effectively inhibit this mutant’s enzymatic activity. However, the KHK^N261A^ mutant completely lost enzymatic activity, suggesting that Asn261 plays a crucial role in the KHK function. Furthermore, when both residues were mutated simultaneously, the enzymatic activity was also abolished (Figure S4E). These findings indicate that Asn261 is likely located within the kinase domain and is essential for the KHK catalytic activity. By binding to this key site, AS-IV effectively inhibits the KHK function, highlighting its regulatory role in fructose metabolism.

**Figure 7:**
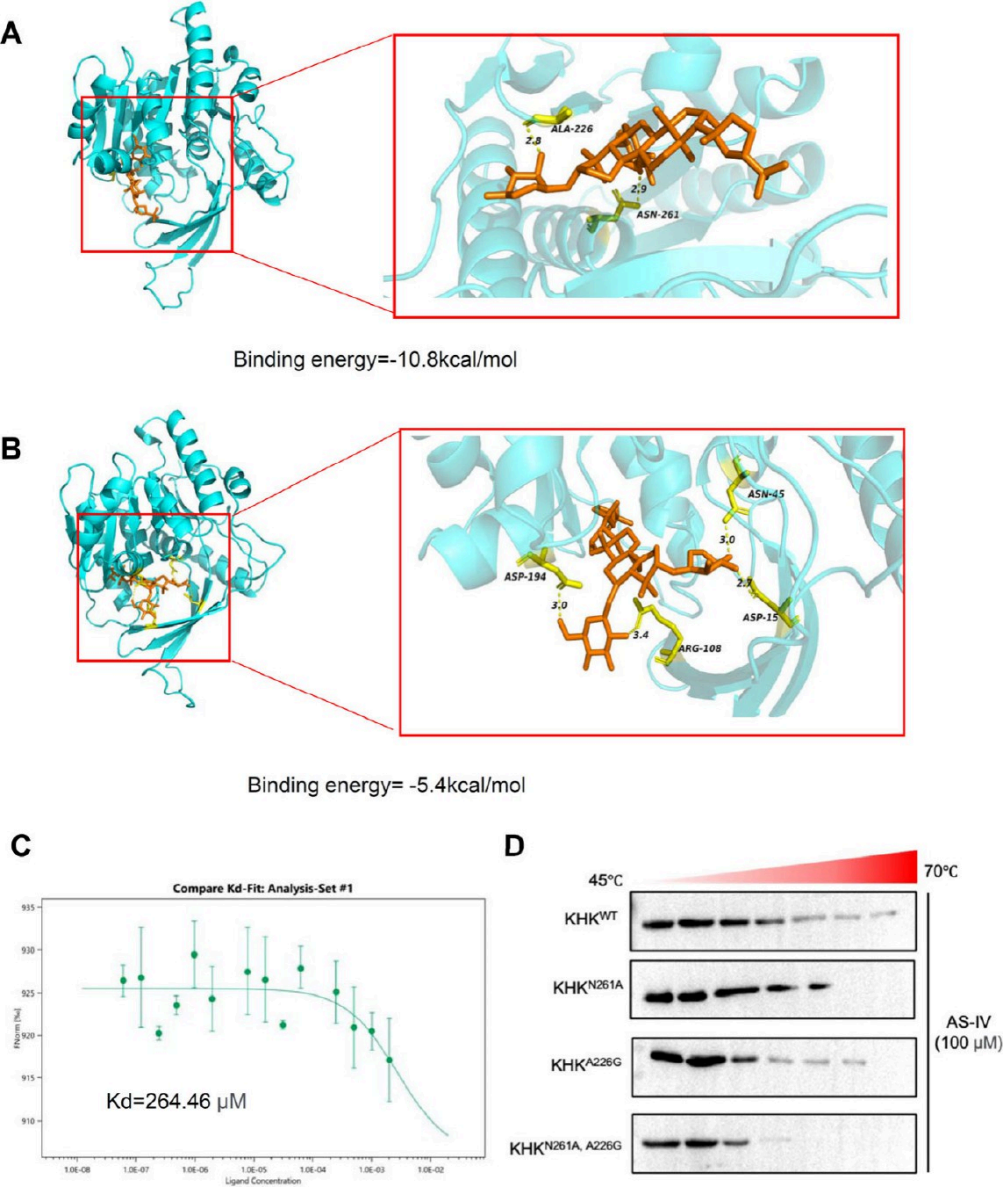
Asn261 and Ala226 in KHK are the critical binding sites for AS-IV. (A) Docking analysis of AS-IV with the KHK protein. (B) Docking analysis of AS-IV with the KHK mutant protein (KHK^N261A, A226G^). (C) Microscale thermophoresis (MST) analysis of AS-IV binding to the KHK mutant protein (KHK^N261A, A226G^), with dissociation constants calculated from three independent replicates. (D) Cellular thermal shift assay (CETSA) assessing the binding of AS-IV to KHK mutant proteins (KHK^N261A^, KHK^A226G^, and KHK^N261A, A226G^) at thermodynamic levels. Data are expressed as mean ± SD (*n* = 3). ‘*’, ‘**’, ‘***’, ‘ns’ indicate significant differences at *P* < 0.05, *P* < 0.01, *P* < 0.001, and no significance levels, respectively.

### 2.7 AS-IV Alleviates Fructose-Induced Intestinal Metabolic Senescence in Mice

To further validate the mechanism by which AS-IV alleviates fructose-induced intestinal metabolic senescence, an intervention study was conducted in mice treated with high-fructose water, followed by AS-IV treatment (Figure 8A). After 8 weeks of AS-IV treatment, a significant inhibition of fructose-induced weight gain was observed (Figure 8B). Jejunum samples were analyzed for SASP markers including inflammatory cytokines *Il6*, *Il1b*, and *Tnf*. AS-IV effectively suppressed the inflammatory response induced by fructose treatment (Figure 8C-E). Histological analysis of the jejunum revealed that fructose exposure significantly reduced the villus height, indicating structural degradation, which was mitigated in AS-IV-treated mice (Figure 8F and 8G). Furthermore, AS-IV inhibited KHK enzyme activity in the intestine, reducing excessive fructose metabolism (Figure 8H).

**Figure 8:**
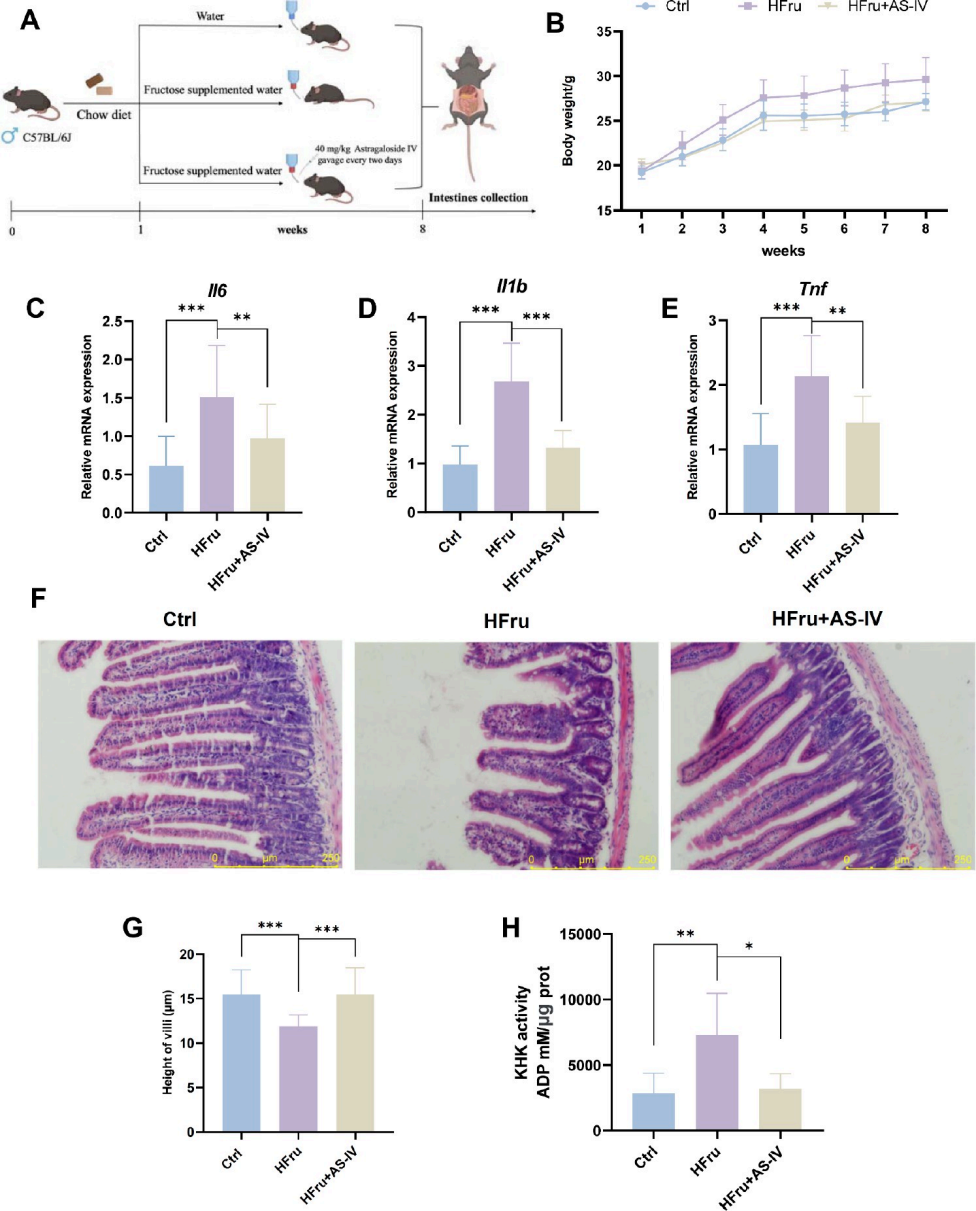
AS-IV alleviates fructose-induced intestinal metabolic senescence in mice. (A) Experimental design and treatment protocol for AS-IV administration in animal models. (B) Body weight of mice. (C-E) RT-qPCR of analysis of inflammatory gene expression associated with SASP (*Il6, Il1b, Tnf*) in mice jejunum. (F) Hematoxylin-eosin staining of the jejunum. (G) Quantitation of jejunal villus length. (H) KHK activity in the jejunum of mice. Data are expressed as mean ± SD (*n* = 6). ‘*’, ‘**’, ‘***’, ‘ns’ indicate significant differences at *P* < 0.05, *P* < 0.01, *P* < 0.001, and no significance levels, respectively.

We further assessed whether AS-IV could restore ISC function impaired by fructose exposure. Immunohistochemical staining for Ki67 and OLFM4 demonstrated enhanced ISC proliferation following AS-IV treatment, accompanied by an increased crypt depth and elevated expression of the ISC marker OLFM4 (Figure 9A–C). Furthermore, AS-IV intervention up-regulated the mRNA expression of ISC markers *Lgr5*, *Olfm4*, and *Ascl2*, suggesting that AS-IV mitigates the adverse effects of fructose on ISC proliferation (Figure 9D). AS-IV also reversed fructose-induced up-regulation of *Trp53*, *Cdkn1a*, and *Rb1* and down-regulation of *Cdk4* and *E2f1*—key cell cycle regulators—at both the mRNA (Figure 9E) and protein levels (Figure 9F and 9G). In summary, these findings indicate that AS-IV mitigates high-fructose-induced intestinal cell cycle arrest and the reduction in ISC numbers, ultimately alleviating fructose-induced intestinal metabolic senescence.

**Figure 9:**
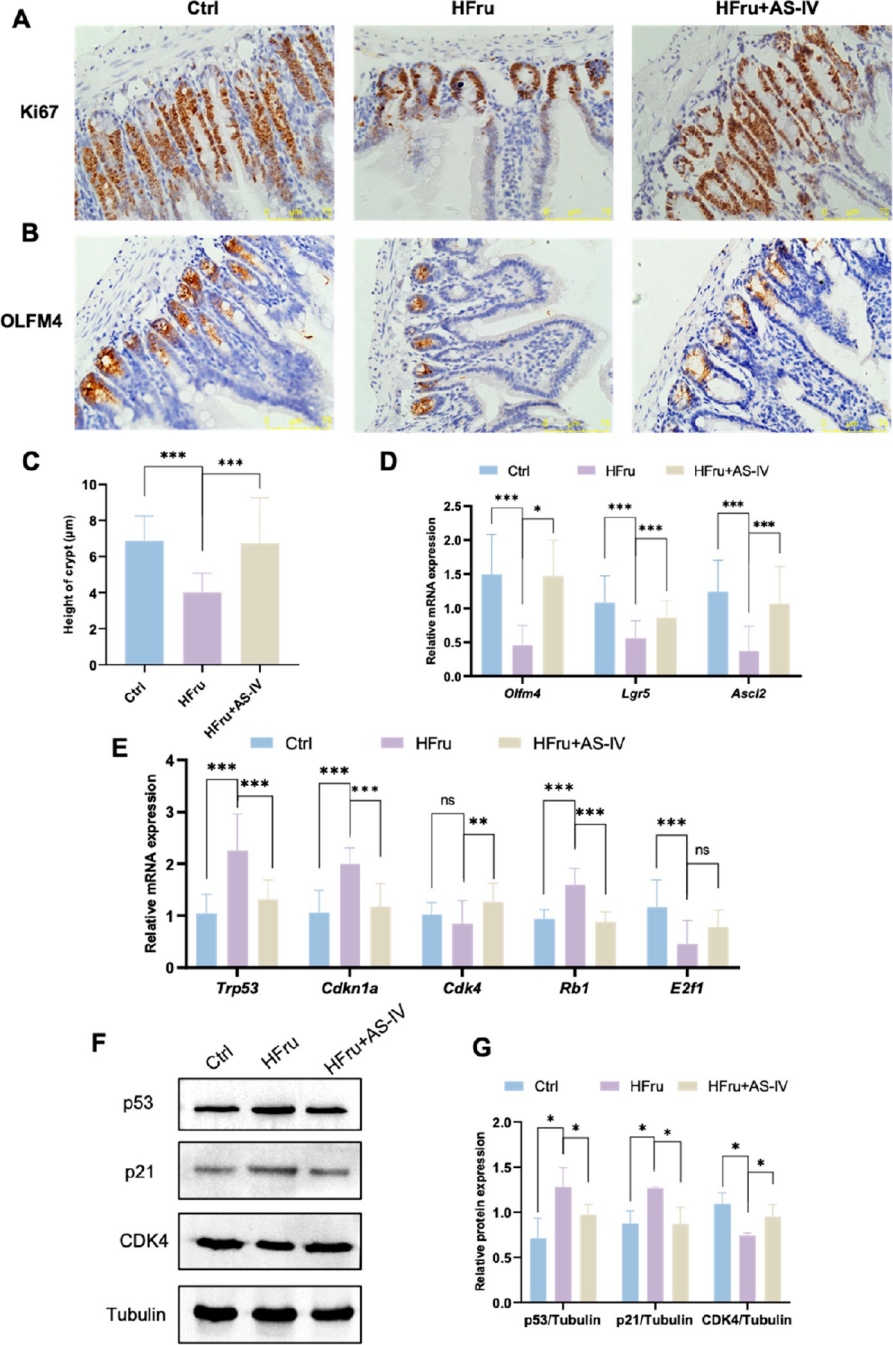
AS-IV alleviates fructose-induced inhibition of intestinal stem cell (ISC) proliferation in mice. (A) Immunohistochemical staining of Ki67 in the jejunum. (B) Immunohistochemical staining of OLFM4 in the jejunum. (C) Quantitation of jejunal crypt height. (D) RT-qPCR of analysis of intestinal stem cell marker (*Olfm4*, *Lgr5*, *Ascl2*) expression in jejunum. (E) RT-qPCR of analysis of cell cycle-related genes (*Trp53*, *Cdkn1a*, *Cdk4, Rb1, E2f1*) expression in the jejunum. (F-G) Representative Western blots and quantitative analyses of indicated protein expression in the jejunum. Data are expressed as mean ± SD (*n* = 6). ‘*’, ‘**’, ‘***’, ‘ns’ indicate significant differences at *P* < 0.05, *P* < 0.01, *P* < 0.001, and no significance levels, respectively.

## 3 Discussion

Metabolic senescence is driven by various factors, including imbalanced energy intake, mitochondrial dysfunction, hyperglycemia, disrupted NAD^+^ metabolism, and nonphysiological oxygen levels.^20^ Recent studies have identified a strong association between excessive fructose consumption and metabolic senescence.^21^ Therefore, elucidating the mechanisms by which dietary fructose contributes to metabolic senescence is critical for developing strategies to delay this process. This study demonstrates for the first time that AS-IV improves high-fructose-induced intestinal senescence. More importantly, the findings reveal that AS-IV directly binds to KHK at Asn261 and Ala226, inhibiting its enzymatic activity and fructose metabolism and preserving intestinal homeostasis. Taken together, these results suggest that AS-IV may serve as a potential therapeutic strategy for mitigating fructose-induced intestinal metabolic senescence.

Fructose, through its unique metabolic pathways, triggers oxidative stress, inflammation, and the accumulation of lipotoxic metabolites, contributing to cellular dysfunction and accelerated aging.^21^ A high-fructose diet induces cellular senescence, increases SA-β-gal activity, and promotes the expression of SASP and senescence markers p53, p21, and p16^3^. Excessive fructose intake promotes ISC dysfunction, leading to a decline in self-renewal capacity and an imbalance in progenitor cell differentiation (unpublished data). These effects disrupt epithelial homeostasis, promote chronic inflammation, and impair nutrient absorption, accelerating the rate of intestinal aging. This study further demonstrates that fructose, along with its metabolic byproducts palmitic acid and ceramide, inhibits ISC proliferation and cell cycle progression, leading to a decline in ISC numbers. Consistent with previous findings, fructose consumption reduces the total number of ISCs, with functional maladaptation to a high-fructose diet arising from disruption of ISC identity, alterations in regional cellular identity, and changes in the composition of mature cell types.^22^ Interestingly, some studies suggest that fructose promotes the proliferation of ISCs, indicating an initial proliferative response to high-fructose intake,^23^ highlighting the complex and bidirectional regulation of ISCs by fructose. It is hypothesized that high-fructose intake initially promotes short-term ISC proliferation as a compensatory mechanism to maintain tissue integrity and function under the increased metabolic stress caused by excessive fructose consumption. This temporary response may facilitate rapid epithelial renewal to counteract the immediate effects of nutrient imbalance and stress. However, prolonged exposure to fructose and its metabolic byproducts disrupts ISC dynamics, impairs epithelial renewal, and ultimately contributes to metabolic senescence. Future studies should investigate the molecular mechanisms governing this transition from ISC proliferation to dysfunction as well as the region-specific effects of fructose along the intestine. A deeper understanding of these mechanisms could aid in the development of targeted interventions to mitigate the adverse effects of excessive fructose consumption on ISC function and delay the onset of intestinal aging.

Natural bioactive compounds offer a promising approach to managing aging. AS-IV, a major active component of *Astragalus membranaceus*, has been shown to alleviate fructose-induced metabolic syndrome in rats.^24^ Furthermore, it suppresses macrophage senescence and M1 polarization while reducing mitochondrial dysfunction and promoting M2 polarization.^25^ This study demonstrates that AS-IV effectively attenuates fructose-induced metabolic senescence by targeting the KHK. KHK catalyzes the conversion of fructose into fructose-1-phosphate, a precursor for fatty acid synthesis and *de novo* lipogenesis.^26,27^ While the current literature mainly attributes these detrimental effects to downstream lipotoxic metabolites such as ceramide and palmitic acid, our findings suggest that fructose-1-phosphate, the immediate product of fructose phosphorylation by KHK, may also play a more direct role. In our intestinal organoid model, F1P supplementation alone was sufficient to induce p53 activation and senescence-associated markers even in the absence of exogenous lipotoxic stress. This observation implies that F1P itself, beyond serving as a precursor for lipid synthesis, may contribute to cellular stress responses and metabolic aging. Although no existing studies have clearly defined a signaling role for F1P in ISC regulation or senescence, our data raise the possibility that fructose metabolism could affect stem cell fate not only through downstream lipotoxicity but also via early metabolites. Future research is warranted to elucidate whether F1P or other intermediates act as metabolic stress signals that directly influence ISC function and intestinal aging. Subsequent steps in fructose metabolism contribute to the accumulation of palmitic acid and ceramide, which are implicated in age-related diseases such as cancer, type 2 diabetes, neurodegeneration, and cardiovascular disorders.^28^ Ceramide accumulation exacerbates mitochondrial dysfunction and disrupts protein homeostasis, accelerating tissue aging.^29^ Our current data presented here demonstrate a clear correlation between increased ceramide levels and elevated senescence markers (p53, p21, SASP). The mechanistic link involving p53 is also supported by previous reports. Notably, previous studies have revealed that specific ceramide species, such as C16-ceramide, can directly regulate p53 signaling. Ceramide binds to a defined site within the DNA-binding domain of p53, stabilizing the protein and preventing its degradation by disrupting the p53–MDM2 interaction. This binding facilitates p53 nuclear translocation and the activation of its downstream targets, thereby promoting a cellular stress response. These findings support our proposed mechanism, in which fructose-induced ceramide accumulation drives cellular senescence via p53 pathway activation. Moreover, our results indicate that AS-IV effectively reduces palmitic acid and ceramide accumulation in the intestine, thus alleviating fructose-induced intestinal metabolic senescence. Collectively, these findings provide new insights into the potential therapeutic applications of AS-IV for preventing or treating age-related metabolic disorders associated with excessive fructose consumption.

Identifying druggable targets is critical for developing therapeutic agents.^30–32^ Several molecular targets of AS-IV have been reported in previous studies. For example, AS-IV promotes angiogenesis and improves cerebral injury following cerebral ischemia by targeting the SIRT7/VEGFA signaling pathway.^33^ Moreover, AS-IV targets AKT1 through the AKT1/GSK-3β signaling pathway to attenuate kidney fibrosis.^34^ Furthermore, AS-IV inhibits PLA2 activity by targeting the catalytic triad of PRDX6, disrupting its interaction with RAC, which impedes NOX2 maturation and reduces oxidative stress damage.^35^ This study identified KHK as a novel target of AS-IV. AS-IV significantly inhibited KHK activity, reducing the conversion of fructose into fructose-1-phosphate and thus limiting the downstream accumulation of metabolites, such as palmitic acid and ceramide. To further confirm this, three target engagement assays were employed, including CETSA, DARTS, and MST, all of which confirm that KHK is a potential target of AS-IV. Fructose metabolism predominantly relies on KHK due to its higher affinity for fructose,^36^ and these findings align with previous studies demonstrating that genetic deletion or pharmacological inhibition of KHK protects against fructose-induced metabolic disorders, including NAFLD/NASH,^37^ type 2 diabetes,^38^ and cardiovascular disease.^39^ These findings highlight KHK as a key metabolic target and establish AS-IV as a promising candidate for combating metabolic senescence induced by excessive fructose intake. The development of AS-IV derivatives with improved pharmacokinetic properties could further improve their efficacy and specificity, providing new opportunities for clinical translation.

The interaction between AS-IV and KHK is mediated by two key residues: Asn261 and Ala226. Molecular docking and MST analyses confirm that these residues play a key role in stabilizing the AS-IV-KHK complex. Asn261 forms a strong hydrogen bond, anchoring AS-IV at the active site of KHK, while Ala226 contributes hydrophobic interactions that complement hydrogen bonding, further stabilizing the complex. These interactions allow AS-IV to precisely position itself at the active site, effectively inhibiting KHK activity and highlighting the importance of these residues in mediating the AS-IV-KHK interaction. The functional significance of Asn261 and Ala226 was further validated through *in vitro* enzymatic activity assays of the KHK mutants. The KHK^A226G^ mutant exhibited reduced enzymatic activity compared with wild-type KHK but retained partial function, and AS-IV remained effective in inhibiting its activity. On the other hand, the KHK^N261A^ mutant completely lost enzymatic activity, highlighting the indispensable role of Asn261 in KHK function. Furthermore, the simultaneous mutation of both residues abolished enzymatic activity entirely, reinforcing that Asn261 resides within the kinase domain and is crucial for KHK’s catalytic function. These findings not only highlight the structural and functional importance of Asn261 but also provide mechanistic insights into how AS-IV effectively suppresses KHK activity by targeting this key regulatory site. Therefore, identifying Asn261 and Ala226 as critical binding residues has significant implications for the design of AS-IV derivatives. Chemical modifications or derivatization of AS-IV could potentially improve its pharmacological properties, such as bioavailability, stability, and tissue targeting, improving its efficacy *in vivo*. For example, introducing moieties capable of forming additional hydrogen bonds or optimizing the hydrophobic profile around Ala226 could improve the overall stability of the AS-IV-KHK complex. Future studies should explore these structural and functional insights to develop optimized therapeutic strategies.

While this study provides strong evidence that AS-IV alleviates intestinal metabolic senescence by targeting KHK, several limitations warrant further investigation. First, the pharmacokinetic properties of AS-IV, including absorption, distribution, metabolism, and excretion, were not explored. These aspects are critical for understanding the therapeutic window, bioavailability, and any potential off-target effects of AS-IV. Future studies should include detailed pharmacokinetic analyses to provide a more comprehensive understanding of AS-IV’s behavior *in vivo*. Furthermore, the long-term safety and potential cumulative effects of AS-IV were not assessed in this study. Longitudinal pharmacokinetic and toxicological studies are essential for establishing a thorough safety profile, which is necessary for the clinical translation of AS-IV as a therapeutic agent. Lastly, further investigations are needed to assess the effects of AS-IV in more complex *in vivo* models and its therapeutic potential in other metabolic diseases. These efforts will help to advance the development of AS-IV-based therapies for metabolic disorders.

## 4 Materials and Methods

### 4.1 Materials and Chemicals

Astragaloside IV (purity ≥ 98%, Cat. No.: 83207–58–3), palmitic acid (purity ≥ 97%, Cat. No.: P101059), and D-fructose 1-phosphate disodium salt (purity ≥ 90%, Cat. No.: 71662–09–4) were obtained from Aladdin (Shanghai, China). D-fructose (purity ≥ 99%, Cat. No.: F108331) was purchased from Sigma (St. Louis, MO, USA). A ceramide mixture (purity: 98.33%, Cat. No.: HY-113679) was obtained from MedChemExpress (Monmouth Junction, NJ, USA). PI/RNase staining buffer (catalog no. 550825) was purchased from BD Biosciences (California, USA). Phospho-Histone H3 (Ser10) antibody (no. 9701), Ki-67 (D3B5) rabbit monoclonal antibody (mAb) (no. 34330), rabbit OLFM4 (D6Y5A) mAb (no. 39141), mouse p53 (1C12) mAb (no. 2524), and rabbit α-tubulin antibody (no. 2144) were obtained from Cell Signaling Technology (Beverly, MA, USA). Anti-p21 antibody (ab109199), anti-CDK4 antibody (ab199728), and anti-ketohexokinase antibody (ab197593) were obtained from Abcam (Cambridge, UK). The ketohexokinase human recombinant protein (NM_000221) was purchased from OriGene Technologies (Rockville, Maryland, USA) The senescence β-galactosidase staining kit (Cat. No.: C0602), HRP-labeled goat antirabbit IgG (H+L) (Cat. No.: A0208), HRP-labeled goat antimouse IgG (H+L) (Cat. No.: A0216), DAPI (Cat. No.: C1005), the BCA protein assay kit (Cat. No.: P0010), and His-tag protein purification kit (Cat. No.: P2226) were purchased from Beyotime (Shanghai, China). DyLight 550-conjugated AffiniPure goat antirabbit IgG (H+L) (Cat. No.: BA1135) was obtained from Boster (Wuhan, China). All other chemical reagents were of analytical grade.

### 4.2 Cell Culture

The rat-derived intestinal epithelial cell line (IEC-6) was obtained from the American Type Culture Collection (ATCC) and cultured in Dulbecco’s modified Eagle’s medium (DMEM; Gibco, New York, USA) supplemented with 10% fetal bovine serum (FBS; Gibco) and 1% penicillin-streptomycin (Gibco) at 37 °C in a humidified atmosphere with 5% CO_2_. Cells were plated in six-well plates at a density of 1 × 10^6^ cells/well and incubated for 24 h. To determine the concentration of fructose required to induce metabolic senescence, cells were treated with 5, 10, or 20 mM fructose for 24 or 48 h. To evaluate the protective effects of AS-IV, cells were exposed to 10 mM fructose alone or in combination with AS-IV at 1, 5, or 10 μM for 48 h. Untreated cells served as the control. At the end of the incubation period, all cells were harvested for further analysis.

### 4.3 *Drosophila melanogaster* Strains and Culture Conditions

The *Drosophila* strains with genotype *W* ^*1118*^ and *esg-Gal4*; *UAS-GFP*; and *tub-Gal80* ^*ts*^ were obtained from the Tsinghua Fly Center (Beijing, China) and were reared at 25 ± 1 °C in a climate control chamber with 60 ± 5% relative humidity under a 12 h light/dark cycle.

Freshly eclosed male flies were reared on a standard sugar-yeast-agar medium for 2 days before being divided into three groups: control (Ctrl), high-fructose diet (HFruD), and high-fructose diet supplemented with AS-IV (HFruD + AS-IV). Flies were reared on the respective diets for 14 days, with medium compositions detailed in Supplementary Table 1. AS-IV was administered at 25, 50, and 100 μM. For *esg-Gal4; UAS-GFP; tub-Gal80* ^*ts*^ flies, the growth temperature was increased to 29 °C on days 13–14 to induce GFP expression in ISC/EB cells.

### 4.4 Senescence β-Galactosidase Stain^40^

Senescence-associated β-galactosidase (SA-β-Gal) activity was measured using a commercial kit according to the manufacturer’s protocol. In brief, IEC-6 cells were washed three times in PBS, fixed for 15 min at room temperature using the fixative solution, and then incubated with fresh SA-β-gal stain solution at 37 °C overnight. Samples were then mounted in 70% glycerol and visualized under a Leica DMI1 microscope (Leica Microsystems, Wetzlar, Germany).

Flies were starved for 6 h and then placed on ice. The guts were dissected in ice-cold PBS, fixed in a fixative solution for 15 min at room temperature, and incubated overnight with SA-β-Gal staining solution at 37 °C. Samples were then imaged using a microscope.

### 4.5 Cell Cycle Analysis

IEC-6 cells were resuspended in ice-cold 70% ethanol and incubated at 4 °C overnight. Cells were then collected by centrifugation, washed with ice-cold PBS, and stained with PI/RNase solution at room temperature for 30 min at a density of 1 × 10^6^ cells/mL. Cell cycle distribution was analyzed using a BD Accuri C6 Plus flow cytometer (BD Biosciences, California, USA), and the proportions of cells in different phases were determined using FlowJo 10.8.1 software.

### 4.6 Immunofluorescence

Flies were starved for 6 h before gut dissection in ice-cold PBS. Dissected guts were immediately fixed in 4% paraformaldehyde for 20 min, followed by three 5 min washes with PBST. Next, the samples were blocked with 0.5% BSA at room temperature for 1 h, followed by overnight incubation with a phospho-histone H3 (Ser10) antibody at 4 °C. After washing, samples were incubated with fluorescent-labeled secondary antibodies for 2 h at room temperature, washed three times with PBST, and stained with DAPI for 10 min. Samples were mounted with 70% glycerol and visualized using a Leica DMI1 microscope (Leica Microsystems, Wetzlar, Germany).

### 4.7 Isolation and Culture of Mouse Intestinal Organoids

Intestinal crypts were isolated from the mice as previously described.^41^ Briefly, a 10 cm segment of the proximal small intestine was excised, washed in ice-cold PBS, and cut into 2 mm pieces to expose the crypts and villi. The tissue was extensively washed (15–18 times) to remove contaminants and then incubated with Gentle Cell Dissociation Reagent (Stem Cell Technologies, Cambridge, MA, USA) at room temperature for 15 min to release crypts. The suspension was then gently filtered through a 70 μm filter to remove debris, and the crypts in the filtrate were collected, counted, embedded in Matrigel (Corning, NY, USA), and cultured in a 24-well plate with IntestiCult organoid growth medium (Stem Cell Technologies) for 10 days until maturity. All experiments were performed by using secondary intestinal organoids. Organoids were treated 72 h after passaging, which allowed sufficient time for recovery and re-establishment of structure and viability.

Organoids were treated for 48 h under three conditions: intestinal organoid growth medium alone, medium with 10 mM fructose, or medium with 10 mM fructose and 5 μM AS-IV. Organoid growth and morphology were monitored throughout the treatment. After treatment, organoids were rinsed with prechilled PBS and collected for further analysis. Palmitic acid and ceramide levels in harvested organoids were measured using biochemical assay kits according to the manufacturer’s protocols.

### 4.8 Treatment of Intestinal Organoids with Ceramide or Palmitic Acid or D-Fructose 1-Phosphate Disodium Salt

Organoids were treated for 48 h under the following conditions: (1) intestinal organoid growth medium alone, (2) medium supplemented with 10 μg/mL ceramide, 100 μM palmitic acid, or 100 μM D-fructose 1-phosphate disodium salt, or (3) medium containing 10 μg/mL ceramide, 100 μM palmitic acid, or 100 μM D-fructose 1-phosphate disodium salt with 5 μM AS-IV. Organoid growth morphology was observed and imaged using a Leica DMI1 microscope.

### 4.9 Organoid Immunofluorescence

Immunofluorescence staining for OLFM4 and Ki67 in the organoids was performed as previously described.^42^ Organoids were cultured in a 24-well plate and treated with intestinal organoid growth medium, medium containing 10 mM fructose, or medium containing 10 mM fructose plus 5 μM AS-IV for 48 h. After treatment, organoids were dispersed by using a gentle cell dissociation reagent and collected by precipitation. The samples were fixed in 4% paraformaldehyde in PBS for 45 min, followed by permeabilization with 1% Triton X-100 for 1 h. Finally, the organoids were incubated with primary antibodies at 4 °C overnight and with secondary antibodies at room temperature for 2 h. Imaging was captured using a Leica DMI1 microscope.

### 4.10 KHK Activity Assay

KHK activity was measured as described previously.^43^ KHK activity levels in intestinal organoids and jejunal tissue were determined by using the ADP-Glo Kinase Assay Kit (V6930, Promega, Wisconsin, USA). Samples were homogenized in tissue lysis buffer (50 mM HEPES, pH 7.4, 150 mM KCl, 4 mM MgCl_2_, 1 mM glutathione, 1 mM EDTA, 1 mM DTT, and 0.05% CHAPS) and centrifuged at 13,000 × *g* for 10 min at 4 °C. Protein concentrations in the supernatant were quantified using the BCA protein assay. KHK activity was measured using 62.5 ng of lysate protein with 1 mM fructose and 100 μM ATP in 1× assay buffer (50 mM HEPES, pH 7.4, 150 mM KCl, 4 mM MgCl_2_, 1 mM glutathione, and 0.05% CHAPS). After 1 h incubation, ADP-Glo reagent and kinase detection reagent were sequentially added at a 40 min interval, and luminescence was measured.

For the in vitro enzyme activity assay of KHK and its mutants, KHK activity was measured using 0.675 ng of purified recombinant human KHK and its mutant proteins with 1 mM fructose and 100 μM ATP in 1× assay buffer (50 mM HEPES, pH 7.4, 150 mM KCl, 4 mM MgCl_2_, 1 mM glutathione, and 0.05% CHAPS). After incubation for 1 h, ADP-Glo reagent and kinase detection reagent were added sequentially at 40 min intervals, and luminescence was subsequently measured.

### 4.11 Mouse Husbandry and Treatment

C57BL/6J mice (6 weeks old) were housed in ventilated cages in a specific pathogen-free (SPF) facility under controlled environmental conditions (temperature: 22 ± 2 °C, relative humidity: 60–70%, 12 h light/dark cycle). The mice had *ad libitum* access to a standard laboratory chow diet and distilled water. All animal experiments were approved by the Animal Care and Use Committee of Wenzhou University (Approval number: WZU-2022–028).

Mice were randomly assigned to three groups (n = 6 per group). The Ctrl group received a standard chow diet and distilled water. The HFru group received a standard chow diet and drinking water containing 20% (w/v) fructose. The HFru + AS-IV group received a standard chow diet and 20% (w/v) fructose in drinking water, along with AS-IV (40 mg/kg) administered via oral gavage every 2 days. The feeding regimen lasted for 8 weeks, after which jejunal tissue was collected for further analyses.

### 4.12 Histology and Immunohistochemistry

Jejunal tissues were fixed in 4% paraformaldehyde overnight, embedded in paraffin, and sectioned into 5-μm-thick slices. Midline sections were stained with hematoxylin and eosin (H&E) for histological analysis. For immunostaining, antigen retrieval was performed by boiling sections in citrate buffer (pH 6) for 10 min, followed by cooling for 60 min at room temperature. Endogenous peroxidase activity was quenched by treating sections with methanol containing 3% H_2_O_2_ for 30 min. Sections were blocked with 1% BSA in PBS at 37 °C for 30 min, followed by overnight incubation at 4 °C with Ki-67 (D3B5) rabbit mAb (no. 34330) (1:400 dilution) or rabbit OLFM4 (D6Y5A) mAb (no. 39141) (1:400 dilution). After being washed, sections were incubated with secondary antibodies (1:50 dilution) for 60 min at 37 °C, washed, and exposed to 3,3′-diaminobenzidine (DAB) chromogen. Counterstaining was performed with hematoxylin for 3 min. Slides were dehydrated, cleared, and permanently mounted by using a xylene-based mounting medium. Tissue morphology was visualized, and images were captured by using a Leica DMI1 microscope (Leica Microsystems).

### 4.13 Drug Affinity Responsive Target Stability (DARTS)

The DARTS assay was performed as described previously.^44^ IEC-6 cells were harvested from two 10 cm culture plates, detached, and lysed in a buffer containing a protease inhibitor cocktail, 1 M sodium fluoride, 100 mM β-glycerophosphate sodium, 50 mM sodium pyrophosphate, 200 mM sodium vanadate, and 690 μL of mammalian protein extraction reagent. Lysates were diluted (1:10) in 10× Tris-NaCl-CaCl_2_ buffer and incubated for 30 min with 10 μM AS-IV, 100 μM AS-IV, or DMSO (control). Following incubation, Pronase (10 mg/mL) was added at a ratio of 1:1000 relative to the protein concentration, and the samples were incubated for 30 min. The reaction was terminated by adding an equal volume of 5× SDS-PAGE loading buffer. Samples were heated at 100 °C for 1 min, subjected to SDS-PAGE, and analyzed by Western blot.

### 4.14 Cellular Thermal Shift Assay (CETSA) and Isothermal Dose–Response Fingerprint (ITDRF_CETSA_)

The identification of the potential targets of AS-IV was performed using CETSA as described previously.^44^ Briefly, IEC6 cells were treated with or without 100 μM AS-IV or DMSO for 2 h. The cell mixture was equally divided into 7 portions and then heated at different temperatures (45–70 °C) for 3 min, followed by cooling on ice. Cells were lysed by repeated freeze–thaw cycles in liquid nitrogen. The lysate was centrifuged, and the supernatant containing the soluble proteins was retained and subjected to Western blot for KHK expression. For the ITDRF_CETSA_ experiments, the procedure was identical to CETSA, except that cells were incubated with increasing AS-IV concentrations (0–100 μM) at 55 °C.

### 4.15 Expression and Purification of KHK Mutant Protein

KHK mutants were cloned into the pET-30a­(+) vector containing an N-terminal His-tag and transformed into *Escherichia coli* BL21 (DE3) competent cells. The plasmid construction and transformation were performed by GenScript Biotech (Nanjing, China). For protein expression, 100 μL of transformed bacteria were inoculated into 10 mL of LB liquid medium containing ampicillin and cultured overnight at 37 °C with shaking at 240 rpm. The next day, 0.5 mL of the overnight culture was transferred into 15 mL of fresh LB medium supplemented with ampicillin and incubated at 37 °C, 240 rpm for 3 h until the optical density at 600 nm (OD_600_) reached 0.4–0.6. Protein expression was induced by the addition of 1 M IPTG to a final concentration of 1 mM, followed by incubation at 37 °C with shaking at 240 rpm for 5 h. Cells were harvested by centrifugation at 800 rpm for 10 min, and the supernatant was discarded. The resulting cell pellets containing the induced proteins were collected. His-tagged proteins were purified using a His-tag protein purification kit (Beyotime, China) according to the manufacturer’s protocol.

### 4.16 Microscale Thermophoresis Analysis (MST)

The binding affinity of KHK and its mutant proteins to AS-IV was measured using MST. His-tagged KHK and its mutant proteins were labeled with the Monolith His-Tag Labeling Kit RED-tris-NTA second Generation (NanoTemper Technologies, Munich, Germany). Serial dilutions of AS-IV (2 mM–60 nM) were mixed with cell lysates containing labeled KHK or mutant proteins at room temperature and loaded into Monolith standard-treated capillaries. Binding measurements were performed by using a Monolith NT.115 instrument (NanoTemper Technologies). Dissociation constants (*K*_d_) were determined using MO. Affinity Analysis software (NanoTemper Technologies).

### 4.17 Molecular Docking

The binding of AS-IV to KHK (PDB ID: 2HQQ) was assessed using AutoDock Vina, which evaluated binding affinity based on binding energy. The three-dimensional structure of AS-IV was retrieved from the PubChem database (https://pubchem.ncbi.nlm.nih.gov/), while the structure of KHK was obtained from the Protein Data Bank (PDB) (http://www.rcsb.org/pdb/home/home.do). A lower binding energy indicated a stronger binding affinity between AS-IV and KHK. The docking results were visualized by using PyMOL.

### 4.18 Molecular Dynamics Simulation

Molecular dynamics simulations were conducted on the KHK-AS-IV complex with the highest docking score using GROMACS 2023.5. The CHARMM36 force field was applied to describe KHK. Periodic boundary conditions were set, and a cubic simulation box extending 1.0 nm from the protein surface was constructed. The system was solvated using the OPC water model, and Na^+^ and Cl^–^ ions were added to balance the negative and positive charges in the system. As initial systems might have structural imperfections, energy minimization was performed using the steepest descent method to rectify any potential erroneous conformations. Then, NVT and NPT equilibrations were carried out with constant temperature at 310 K and constant pressure at 1 bar, with each phase lasting 100 ps. The complexes then underwent an 80 ns simulation, with data recorded every 2 fs.

### 4.19 Quantitative RT-PCR

Total RNA was extracted from the IEC6 cells, *Drosophila* guts, intestinal organoids, or mouse jejunum tissues using TRIzol reagent (Invitrogen, Carlsbad, CA, USA). cDNA was synthesized from the extracted RNA using a reverse transcription kit (Takara, Shiga, Japan) according to the manufacturer’s protocol. Quantitative PCR (qPCR) was then performed using the SYBR Green PCR Master Mix (Applied Biosystems, Foster City, CA, USA). All primers used in this study are listed in Supplementary Table 2. Gene expression levels were normalized to *Rp49* in *Drosophila* and *Actb* in IEC-6 cells, intestinal organoids, and mice. The transcript levels were calculated by using the 2^–ΔΔct^ method.

### 4.20 Western Blotting

Cell lysates, intestinal organoids, and mouse intestinal tissue extracts were prepared using RIPA buffer (Beyotime, Nantong, China). Protein samples (20 μg) were separated by 12% SDS-PAGE and transferred to a PVDF membrane (Millipore, Burlington, USA). The membrane was blocked with 5% skim milk and incubated overnight at 4 °C with the following primary antibodies (all at 1:1000 dilution): mouse p53 (1C12) mAb (no. 2524), rabbit α-tubulin antibody (no. 2144), anti-p21 antibody (ab109199), anti-CDK4 antibody (ab199728), or antiketohexokinase antibody (ab197593). After washing, the membrane was incubated with appropriate secondary antibodies at room temperature for 2 h, followed by further washing. Protein bands were detected using enhanced chemiluminescence autoradiography (Thermo Fisher Scientific, Waltham, USA) and visualized using a Tanon 4800 Multisystem (Tanon, Shanghai, China).

### 4.21 Statistical Analysis

Data were analyzed using GraphPad Prism 9 (GraphPad Software Inc., La Jolla, CA, USA). Statistical differences between two groups were assessed using Student’s *t*-test, while comparisons among more than two groups were conducted using one-way ANOVA followed by Tukey’s multiple comparisons test. *P* values of less than 0.05 were considered statistically significant.

## 5 Conclusions

In summary, this study demonstrates the protective effects of AS-IV against high-fructose-induced intestinal metabolic senescence and identifies KHK as its molecular target. The interaction between AS-IV and KHK is stabilized by Asn261 and Ala226, and inhibition of KHK activity reduces the accumulation of fructose metabolites including palmitic acid and ceramide. This intervention restores normal cell cycle dynamics in intestinal stem cells, mitigates SASP-associated inflammation, and alleviates pathological features of cellular senescence. These findings provide strong evidence for AS-IV as a potential therapeutic agent for fructose-induced metabolic senescence and related metabolic disorders.

## Supplementary Material



## Data Availability

Data will be made available on request.

## Notes

Effects of Astragaloside IV treatment, SA-β-gal activity, SASP expression analysis. Molecular docking and MST analysis of Astragaloside IV’s target, KHK enzymatic activity assay, composition of *Drosophila* culture medium, and primer sequences for quantitative PCR (PDF)
