# Supplementary material for: Astragaloside IV Alleviates Fructose-Induced Intestinal Metabolic Senescence by Targeting Ketohexokinase Asn261/Ala226 to Preserve Intestinal Stem Cell Homeostasis
Source: ACS Cent Sci. 2025 Jul 29;11(9):1682–99. doi: 10.1021/acscentsci.5c00726 (PMC12464770; doi:10.1021/acscentsci.5c00726)
Supplement: Supplementary file 1 [file oc5c00726_si_001.pdf]

Supplementary Materials for

Astragaloside IV Alleviates Fructose-Induced Intestinal Metabolic Senescence by Targeting Ketohexokinase Asn261/Ala226 to Preserve Intestinal Stem Cell Homeostasis

Qifang Wu [a], Yingna Li [a], Yunyun Zhao [d], Ruifen Zhang [c], Jingyang Tong [c], Chunlei Ji [a], Yiming Zhao [a], Mingjiang Wu [c], Xiaosheng Jin [e], Dandan Wang [a,*], Haibin Tong [c,*], Liwei Sun [a,*], Fangbing Liu [b,*]

[a] *Research Center of Traditional Chinese Medicine, College of Traditional Chinese Medicine, Changchun University of Chinese Medicine, Changchun 130021, China.*

[b] *Northeast Asian Institute of Traditional Chinese Medicine, Changchun University of Chinese Medicine, Changchun 130021, China.*

[c] *College of Life and Environmental Science, Wenzhou University, Wenzhou 325035, China;*

[d] *Department of Endocrinology and Metabolism, Affiliated Hospital to Changchun University of Chinese Medicine, Changchun 130021, China*

[e] *Department of Gastroenterology, The Third Affiliated Hospital of Wenzhou Medical University, Wenzhou 325200, China*

*Corresponding author:

E-mail address: liufb@ccucm.edu.cn (F.L.); sunnyliwei@ccucm.edu.cn (L.S.); tonghaibin@gmail.com, tonghb@wzu.edu.cn (H.T.); wangdandan2171@163.com (D.W.).

This file includes:

Figure S1 Fructose-induced metabolic senescence in IEC-6 cells

Figure S2 AS-IV alleviates fructose-induced increase in SA-β-gal activity and SASP markers expression in IEC-6 cells

Figure S3 AS-IV alleviates fructose-induced increase in SA-β-gal activity and SASP markers expression in the *Drosophila* gut

Figure S4 KHK mutation attenuates its binding affinity to AS-IV

Table S1 Composition of *Drosophila* culture medium

Table S2 Primer sequences for quantitative PCR

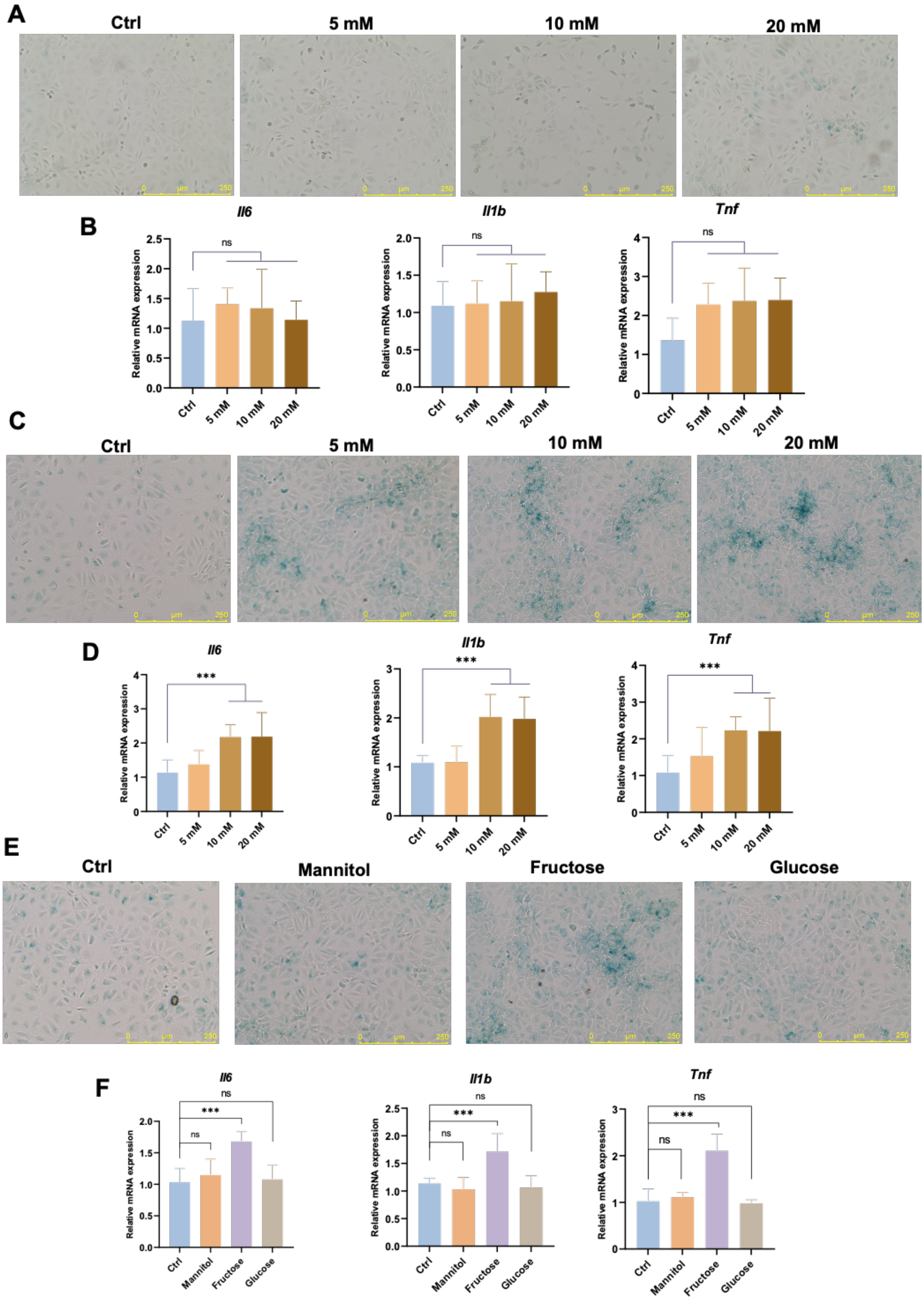

Figure S1. Fructose-induced metabolic senescence in IEC-6 cells. (A) SA-β-gal staining of IEC-6 cells treated with fructose 24 h. (B) RT-qPCR of analysis of inflammatory gene expression associated with the SASP, including *Il6*, *Il1b*, and *Tnf*, in IEC-6 cells treated with fructose 24 h. (C) SA-β-gal staining of IEC-6 cells treated

with fructose 48 h. (D) RT-qPCR of analysis of inflammatory gene expression associated with the SASP, including *Il6*, *Il1b*, and *Tnf*, in IEC-6 cells treated with fructose 48 h. (E) SA-β-gal staining of IEC-6 cells treated with mannitol or fructose or glucose 48 h. (F) RT-qPCR of analysis of inflammatory gene expression associated with the SASP, including *Il6*, *Il1b*, and *Tnf*, in IEC-6 cells treated with mannitol or fructose or glucose 48 h. Data are expressed as mean ± SD (n = 3). '*', '**', '***', 'ns' indicate significant differences at $P < 0.05$, $P < 0.01$, $P < 0.001$, and no significance levels, respectively.

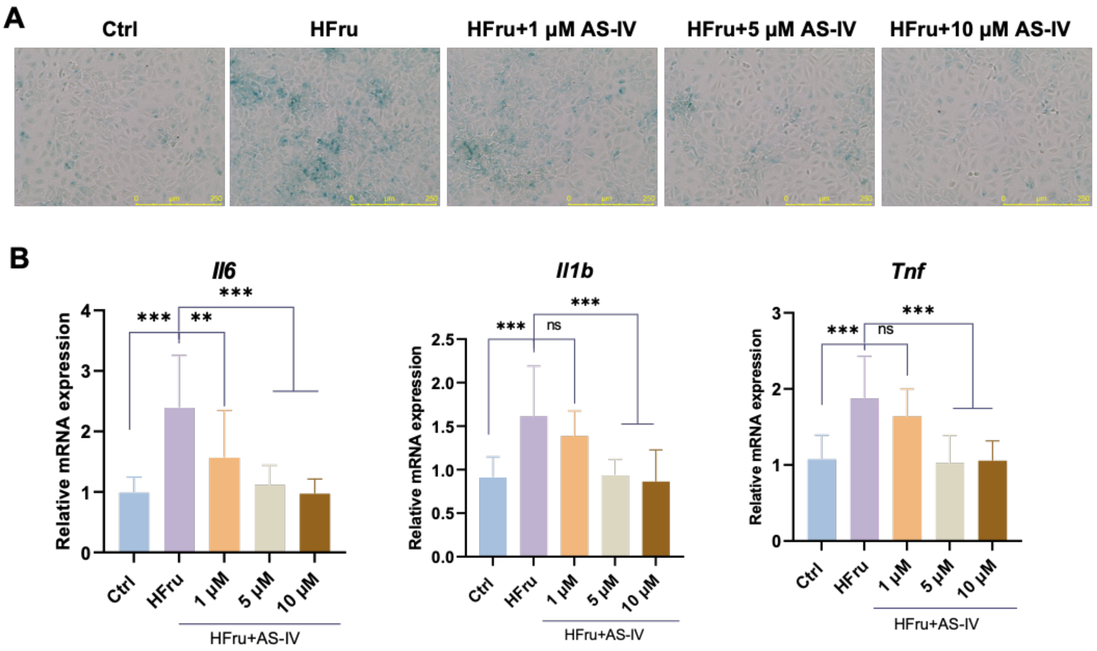

Figure S2. AS-IV alleviates fructose-induced increase in SA-β-gal activity and SASP markers expression in IEC6 cells. (A) SA-β-gal staining of IEC6 cells. (B) RT-qPCR of analysis of inflammatory gene expression associated with the SASP, including *Il6*, *Il1b*, and *Tnf*, in IEC6 cells. Data are expressed as mean ± SD (n = 3). '*', '**', '***', 'ns' indicate significant differences at $P < 0.05$, $P < 0.01$, $P < 0.001$, and no significance levels, respectively.

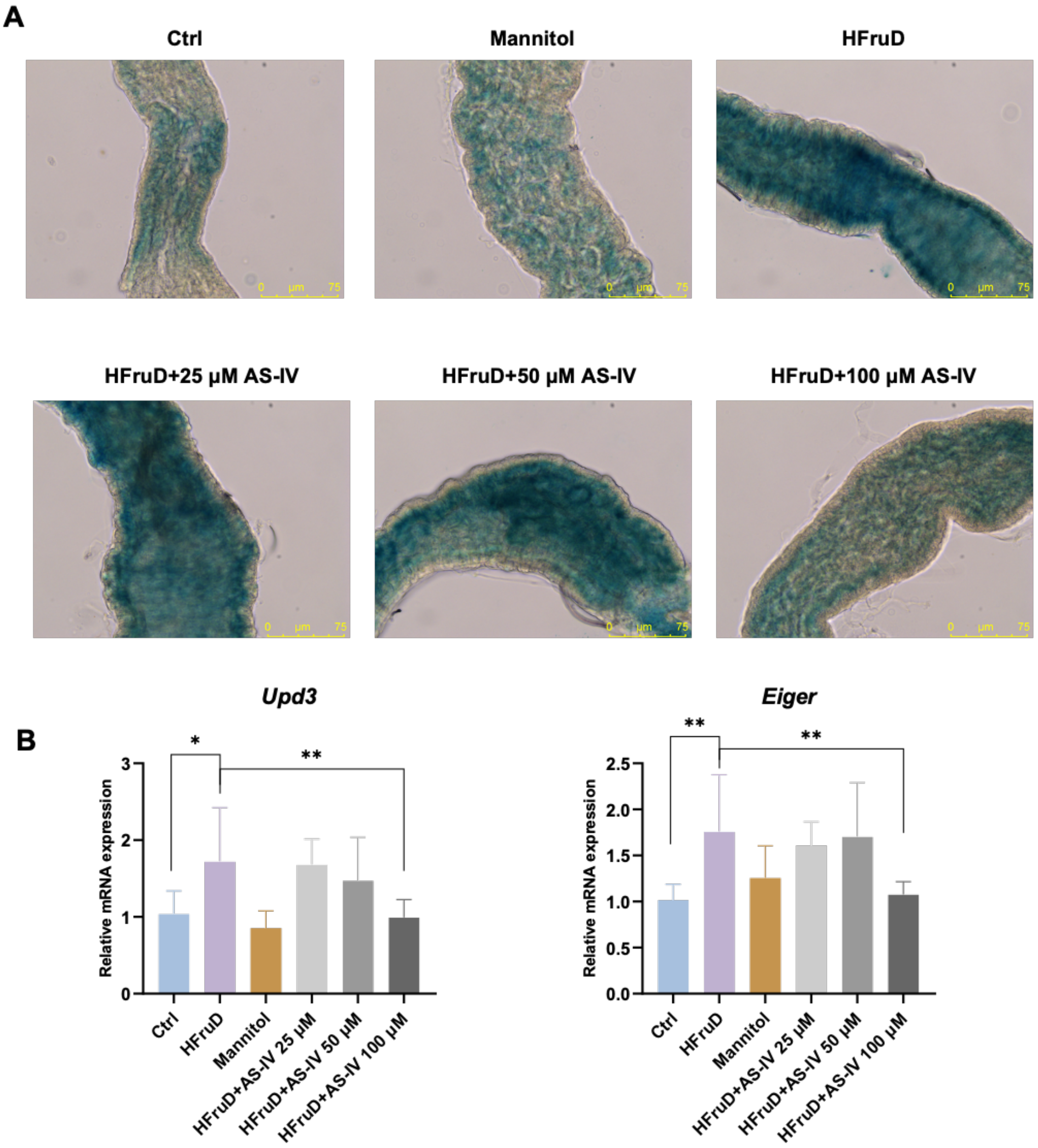

Figure S3. AS-IV alleviates fructose-induced increase in SA-β-gal activity and SASP markers expression in the *Drosophila* gut. (A) SA-β-gal staining of *Drosophila* gut. (B) RT-qPCR of analysis of inflammatory gene expression associated with the SASP, including *Upd3* and *Eiger*, in *Drosophila* gut. Data are expressed as mean ± SD (n = 3). '*', '**', '***' indicate significant differences at $P < 0.05$, $P < 0.01$, and $P < 0.001$ levels, respectively.

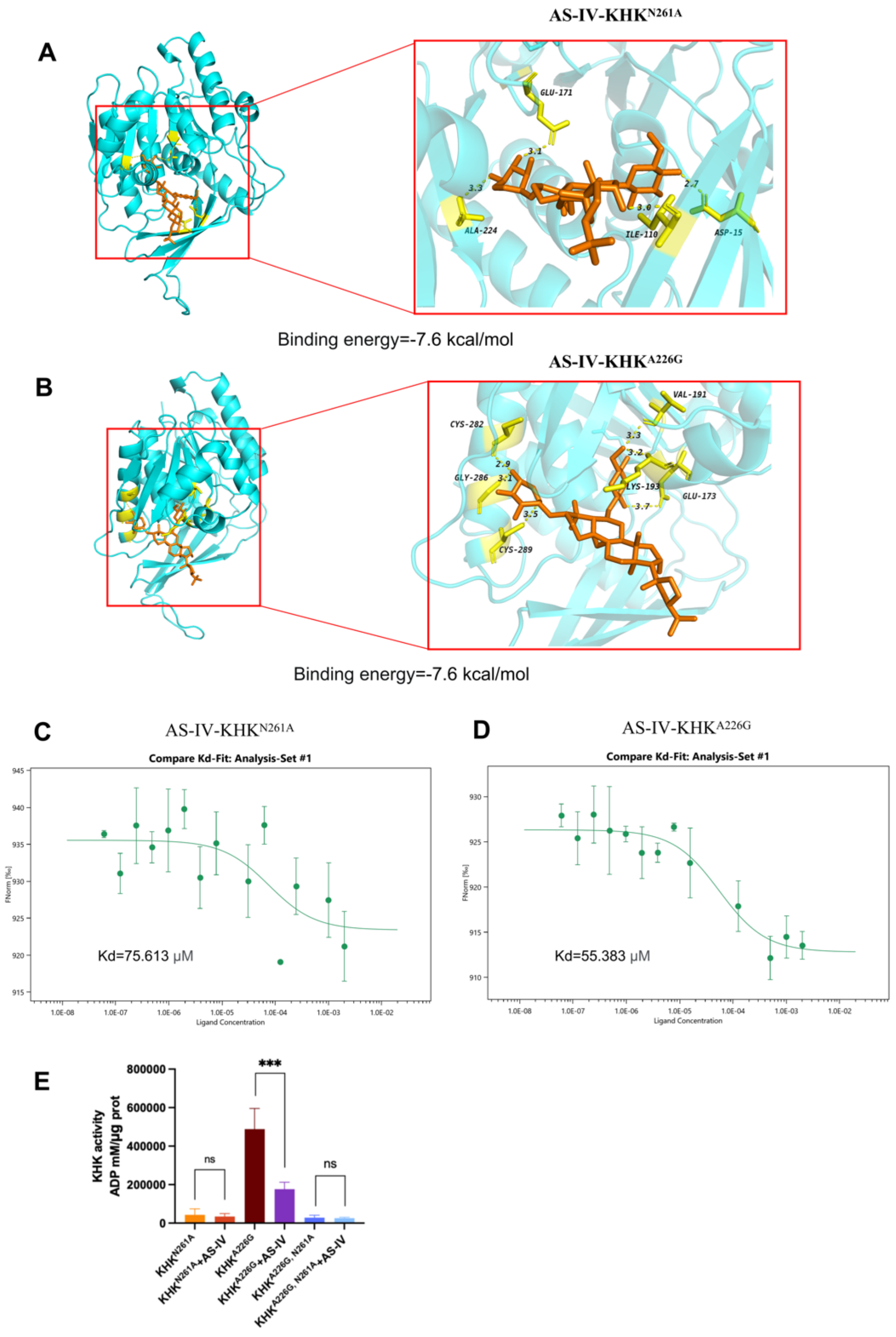

Figure S4. KHK mutation attenuates its binding affinity to AS-IV. (A) Docking analysis of AS-IV with KHK^{N261A} protein. (B) Docking analysis of AS-IV with KHK^{A226G} protein. (C) Microscale thermophoresis analysis (MST) of AS-IV binding to KHK^{N261A} protein. The dissociation constants were calculated by three independent replicates. (D) Microscale thermophoresis analysis (MST) of AS-IV binding to

KHK^{A226G} protein. (D) KHK mutant protein activity inhibition experiment. The dissociation constants were calculated by three independent replicates Data are expressed as mean ± SD (n = 3). '*', '**', '***', 'ns' indicate significant differences at $P < 0.05$, $P < 0.01$, $P < 0.001$, and no significance levels, respectively.

Table S1. Composition of *Drosophila* culture medium

***Drosophila* Culture medium**	**Ingredients**	**Content**
**Control (Ctrl)**	sucrose	5 g
	agar	0.7 g
	corn starch	8 g
	yeast	3.2 g
	propionic acid	400 μL
	nipagin	0.05 g
	water	100 mL
**High fructose diet (HFruD)**	sucrose	5 g
	fructose	20 g
	agar	0.7 g
	corn starch	8 g
	yeast	3.2 g
	propionic acid	400 μL
	nipagin	0.05 g
	water	100 mL
**High fructose diet +AS-IV (HFruD+AS-IV)**	sucrose	5 g
	fructose	20 g
	agar	0.7 g
	corn starch	8 g
	yeast	3.2 g
	propionic acid	400 μL
	nipagin	0.05 g

	water	100 mL
	AS-IV	25/50/100 μM

Table S2. Primer sequences for quantitative PCR

Gene	Forward (5' to 3')	Reverse (5' to 3')
Drosophila		
Upd3	TGCCCTAGGAAAAGCGATCTG	ATCACGCTGGAGTTGTATCTCG
Eiger	GGCAGTTTGTCGCCTGATG	CAGACTCCTGTCCAAGAGCTGTT
P53	CCCATCCAACCACTTAATTTGCG	AAGGTGATTTTGACAGCGGAC
Dap	GGTACAGCCAACAAAACACCCT	TTTCAGCTAAAGGGCAGCCG
Cdk4	ACAGTTTCCAGAGCTGTGCC	GGGACATTTTCTGCCGCTTC
Rbf	GGAGCTGGACTTTCGTCACA	TTGCCGTAGATGGTCGAAGC
E2f1	CTTGGAACATCGGGCGAAGA	ACGCTCATTCGTATGGCTCG
Rp49	GACAGTATCTGATGCCCAACA	CTTCTTGGAGGAGACGCCGT
Mus		
Il6	ACAAAGCCAGAGTCCTTCAGAG	GTGACTCCAGCTTATCTCTTGG
Il1b	GCCACCTTTTGACAGTGATGAG	AAAGGTTTGGAAGCAGCCCT
Tnf	ACCCTCACACTCACAAACCA	TGTGGGTGAGGAGCACGTA
Lgr5	TGCCATCTGCTTACCAGTGT	GGTCCCGCTCATCTTGAACT
Olfm4	CATGCTCGAAGTGGAGATAAGG	CGGCGAATGCTAAGGACATTG
Ascl2	GAGAGCTAAGCCCGATGGAG	CCAGGGATGCAGCTTAGGG
Trp53	TCCGAAGACTGGATGACTGC	GATCGTCCATGCAGTGAGGT
Cdkn1a	GCAGAATAAAAGGTGCCACAGG	AAAGTTCCACCGTTCTCGGG
Cdk4	TAGCCGAGCGTAAGGCTGAT	CCAGGCCGCTTAGAAACTGA
Rb1	CCCTTGCATGGCTTTCAGATTC	AGGACAAGCAGGTTCAAGGT
E2f1	GACTGCCTTGCCTGTCTGTT	GTGCACTAAGCAAGCACCAG
Actb	ACACCCGCCACCAGTTCG	GAGTCCTTCTGACCCATTCCC
IEC6 (Rat)		

Actb	CCCGCGAGTACAACCTTCTTG	GTCATCCATGGCGAACTGGTG
Il6	GCCCACCAGGAACGAAAGTC	TGGCTGGAAGTCTCTTGCGG
Il1b	TGTCTGAAGCAGCTATGGCA	ACAGGTCATTCTCCTCACTGTC
Tnf	ATGGGCTCCCTCTCATCAGT	GCTTGGTGGTTTGCTACGAC
Trp53	CCCCTGAAGACTGGATAACTGT	AACTCTGCAACATCCTGGGG
Cdkn1a	TGTGATATGTACCAGCCACAGG	CGAACAGACGACGGCATACT
Cdk4	GCCGAGCGTAAGGCTGAT	CGCTTAGAAACTGGCGCATC
Rb1	TGGAAGCCAACTTGACAAGAGA	CAAACAAAGGTGAATCAGAAAGCC
E2f1	ACGTGCTGCTCTTCGCAAC	AGCCTCCGTTTCACCGGC
